# Detecting major introgressions in wheat and their putative origins using coverage analysis

**DOI:** 10.1038/s41598-022-05865-w

**Published:** 2022-02-03

**Authors:** Jens Keilwagen, Heike Lehnert, Thomas Berner, Ekaterina Badaeva, Axel Himmelbach, Andreas Börner, Benjamin Kilian

**Affiliations:** 1 Julius Kuehn Institute, Quedlinburg, Germany; 2 N.I. Vavilov Institute of General Genetics, Russian Academy of Sciences, Moscow, Russia; 3 The Federal Research Center Institute of Cytology and Genetics, Siberian Branch of the Russian Academy of Sciences (ICG SB RAS), Novosibirsk, Russia; 4 Leibniz Institute of Plant Genetics and Crop Plant Research (IPK), Gatersleben, Germany; 5 Global Crop Diversity Trust, Bonn, Germany

**Keywords:** DNA sequencing, Genome informatics, Plant breeding, Agricultural genetics, Plant hybridization

## Abstract

Introgressions from crop wild relatives (CWRs) have been used to introduce beneficial traits into cultivated plants. Introgressions have traditionally been detected using cytological methods. Recently, single nucleotide polymorphism (SNP)-based methods have been proposed to detect introgressions in crosses for which both parents are known. However, for unknown material, no method was available to detect introgressions and predict the putative donor species. Here, we present a method to detect introgressions and the putative donor species. We demonstrate the utility of this method using 10 publicly available wheat genome sequences and identify nine major introgressions. We show that the method can distinguish different introgressions at the same locus. We trace introgressions to early wheat cultivars and show that natural introgressions were utilised in early breeding history and still influence elite lines today. Finally, we provide evidence that these introgressions harbour resistance genes.

## Introduction

Wheat (*Triticum aestivum*, 2n = 6x = 42, BAD) is one of the most widely grown^1^ and consumed crops in the world^2^. Due to expected population growth and declining acreage for crop cultivation, projections indicate that the wheat yield per hectare will need to increase significantly over the next decades^3^. More frequent and severe abiotic and biotic stresses will also affect crop yield^4,5^, so breeding for yield stability in wheat is becoming more important^6^. As a result of rapid advances in sequencing technologies combined with decreasing costs, the first complete wheat genome has been sequenced^7^ and the first step towards a wheat pan-genome sequence has been made^8^. These resources, combined with high-density SNP matrices and high-throughput phenotyping, allow for the identification of genes related to specific traits and the improvement of breeding strategies.

Traditional breeding strategies, which are based on crossing elite cultivars and selecting the best offspring, have increased yield, but have decreased the genetic diversity of crop plants compared with that of their CWRs^9^. Interspecific hybridisation, also known as interspecies crossing, wide hybridisation, or distant hybridisation, allows the transfer of DNA from a donor species into a crop plant. This strategy has been used in breeding to add desired traits to crop plants, for example, resistance or tolerance to biotic or abiotic stress^10–12^. The foreign DNA that is derived from the donor species and has been integrated into the genome is called an introgression. Traditionally, large introgressions have been detected using cytological methods^13–16^, but these methods are low-throughput.

Fortunately, the wealth of wheat reference quality assemblies (RQAs)^8^ and next generation sequencing (NGS) data for CWRs make it possible to investigate interspecific hybridisation events without cytological methods. Analyses of SNPs can be used to detect introgressed regions in crop plants^17,18^. If the donor is unknown, coverage analysis or transposable element analysis can be used to detect candidate regions of introgressions^8,19^. Such computational methods can be used to trace introgressed regions in breeding materials, and this information can be used in breeding programs to minimise such regions to increase wheat yield and yield stability.

However, the methods mentioned above do not identify putative donor species, which is especially interesting for elite lines with multiple introgressions from different donors or for old landraces. For such wheat accessions, the origin of a genomic region that might influence an important trait is often unknown. This also hampers the search for additional beneficial alleles. Here, we describe a method for identifying introgressions and predicting putative donor species and demonstrate its applicability using 10 RQAs of wheat.

## Results

### Detection of nine introgressions in 10 wheat RQAs

We aimed to detect introgressions by mapping publicly available short reads to wheat reference genomes^8^ (Table S1). We identified putative introgressions as regions with decreased coverage of reads from the progenitor species of the wheat subgenomes; that is, for a genomic window, the proportion covered by reads is decreased (Methods). *Triticum urartu* (2n = 2x = 14, A^u^) and *Aegilops tauschii* (2n = 2x = 14, D) are the donors of the A and D subgenomes of wheat^20–22^, respectively, whereas the donor of the B subgenome is either not known yet or extinct^23^. *Aegilops speltoides* (2n = 2x = 14, S) is the most closely related remnant taxon to the wheat B subgenome donor^23–25^. For this reason, decreased coverage of reads from the subgenome donors can be expected at introgressed regions of the A and D subgenomes, but not for the B subgenome using *Ae. speltoides* as the subgenome donor. Increased coverage of reads from a wild relative in the same region indicates that it is a putative donor species of this introgression. Because several wheat relatives share a common ancestor or were subgenome donors for polyploid *Triticum* and *Aegilops* taxa, increased coverage of reads from a wheat relative at an introgressed region in an elite background provides hints about the source of the introgression, but does not necessarily identify the exact donor species.

**Figure 1:**
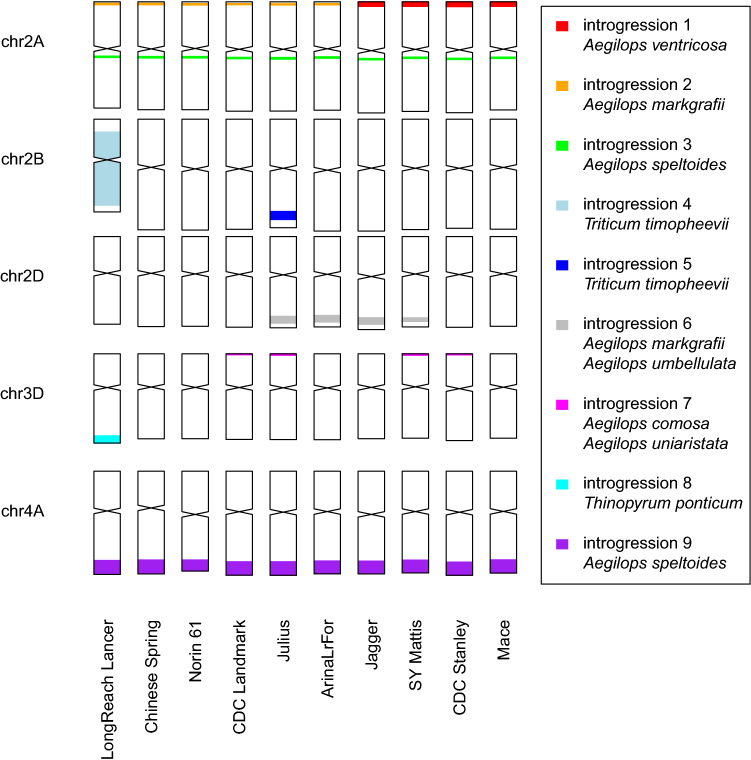
Overview of detected introgressions and putative donor species in the 10 wheat reference quality assemblies (RQAs).

Using this method and a wide range of wheat relatives^8,26,27^ (Table S2), we detected nine large introgressions in the 10 wheat RQAs, located on chromosomes 2A, 2B, 2D, 3D, and 4A (Fig. 1, Table S3, Supplementary Data 1). For all nine introgressions, we identified a putative donor.

**Figure 2:**
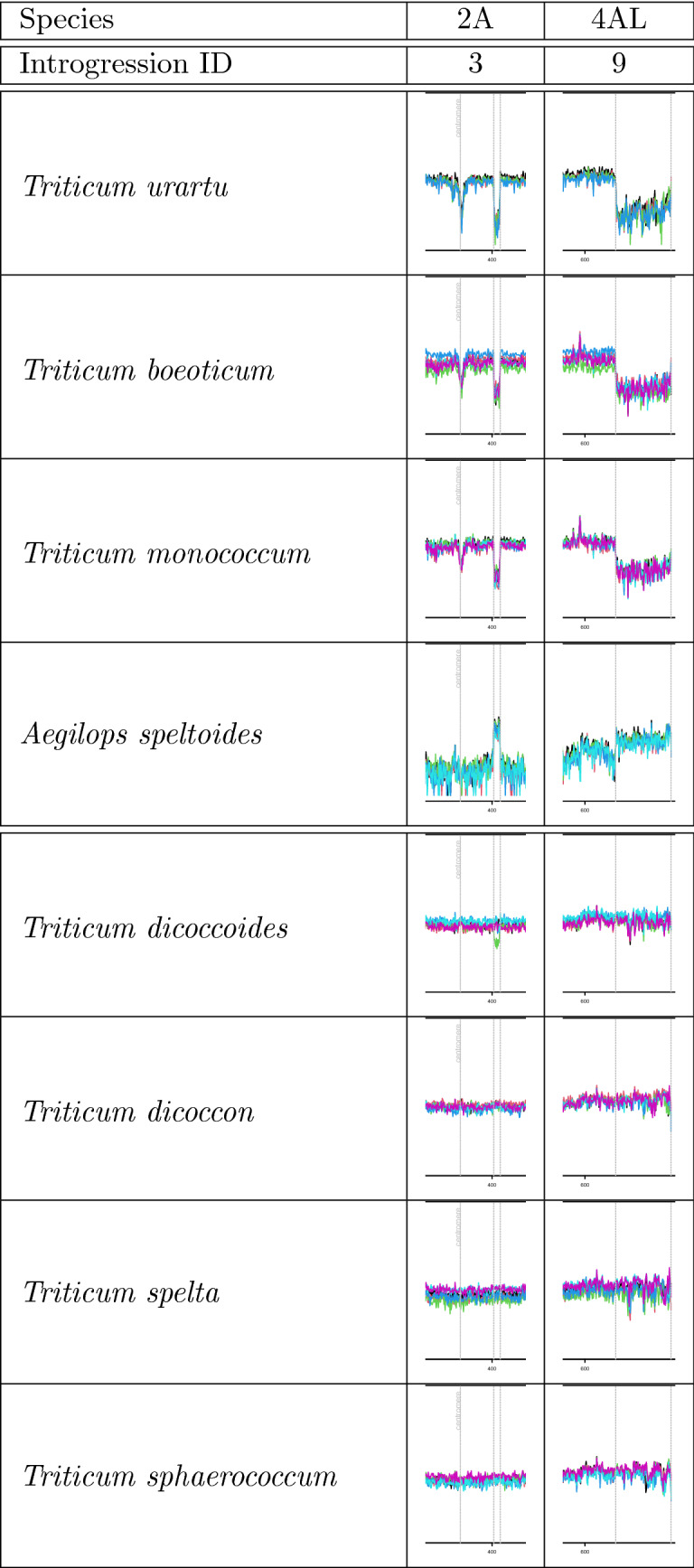
Old introgression events on chromosomes 2A (introgression 3) and 4AL (introgression 9) shown for cv. Julius as reference. Different colours indicate different accessions used for each species, but the colours are of minor interest since almost all accessions show the same profiles. We detected regions with low coverage for the A-subgenome donor *Triticum urartu* and increased coverage for the putative donor *Aegilops speltoides*. Interestingly, we also detected these low-coverage regions in *Triticum boeoticum* and *Triticum monococcum*, which carry only the A genome. In contrast, these regions showed normal coverage in the other polyploid wheat relatives carrying at least A and B subgenomes, indicating that these relatives harbour a sequence similar to that in the reference sequence of cv. Julius. The only exception is the *Triticum dicoccoides* accession K240104 (indicated by green line), which also harbours a low-coverage region on chromosome 2A.

Interestingly, two introgressions were detected in all 10 RQAs; one was close to, but downstream of the centromere of chromosome 2A (introgression 3) and the other was on chromosome 4AL (introgression 9). On the one hand, we detected regions with decreased coverage on chromosomes 2A and 4AL in *Triticum urartu*, *Triticum boeoticum* (2n = 2x = 14, A^b^) and *Triticum monococcum* (2n = 2x = 14, A^b^) indicating that these three species share similar sequences in these regions of 2A and 4AL. On the other hand, normal coverage in these regions was detected in polyploid relatives including *Triticum dicoccoides* (2n = 4x = 28, BA), *Triticum dicoccon* (2n = 4x = 28, BA), *Triticum spelta* (2n = 6x = 42, BAD), and *Triticum sphaerococcum* (2n = 6x = 42, BAD) (Fig. 2), suggesting that these species share a similar sequence compared to the reference sequence of cv. Julius. This introgressions might be common to all these polyploid taxa. One exception was accession K240104 of *T. dicoccoides* originating from Syria^27^, in which a low-coverage region was detected on chromosome 2A. Based on the assumption that *Triticum urartu* is the A-subgenome donor and *Aegilops speltoides,* which shows increased coverage in these regions, is the B-subgenome donor, we might speculate that these introgressions might date back to the time of hybridization between A and B subgenome.

The low-coverage region on chromosome 2A is located at about 395–407 Mb in Chinese Spring and harbours 17 annotated protein-coding genes (TraesCS2A01G257500–TraesCS2A01G259100). Upstream of this introgression is another small low-coverage region overlapping with the position of the centromere^8,26,27^. Similar small low-coverage regions overlapping with the centromere were also detected on chromosomes 1A to 5A.

Chromosome 4A is structurally highly rearranged and has retained a large portion of chromosome 7BS through a species-specific translocation^28,29^. Studies using cytological and NGS methods have clearly shown the rearrangement on 4AL^30,31^. Interestingly, we did not detect regions with increased coverage of reads from wheat chromosome 7B in *T. urartu, T. boeoticum,* or *T. monococcum*. Since both introgressions (introgressions 3 and 9) are very old and were found in all wheat RQAs as well as in the polyploid CWRs, they were not treated as introgressions in a recent RQA analysis^8^.

We also detected four previously described introgressions. Firstly, two introgressions on chromosome 2B and 2BL (introgressions 4 and 5), potentially originating from *Triticum timopheevii* (2n = 4x = 28, GA^t^), were detected in cv. LongReach Lancer and cv. Julius, respectively. Both cultivars are descendants of cv. Wisconsin-245^8,32^, which has an introgression of the nearly complete chromosome 2G of *T. timopheevii* (Fig. S1). This introgression harbours the resistance gene *Sr36* on chromosome 2BS^33,34^. Interestingly, cv. LongReach Lancer has a long introgression spanning chromosome 2BS and 2BL^8^, whereas cv. Julius harbours a much smaller introgression on chromosome 2BL that, to the best of our knowledge, has not been previously associated with *T. timopheevii*.

Secondly, an introgression was detected only in cv. LongReach Lancer on chromosome 3DL with the putative donor *Thinopyrum ponticum* (2n = 10x = 70, EEE^St^E^St^) (introgression 8). A previous study of cv. LongReach Lancer detected an introgression on chromosome 3DL from *Th. ponticum*^8^.

**Figure 3:**
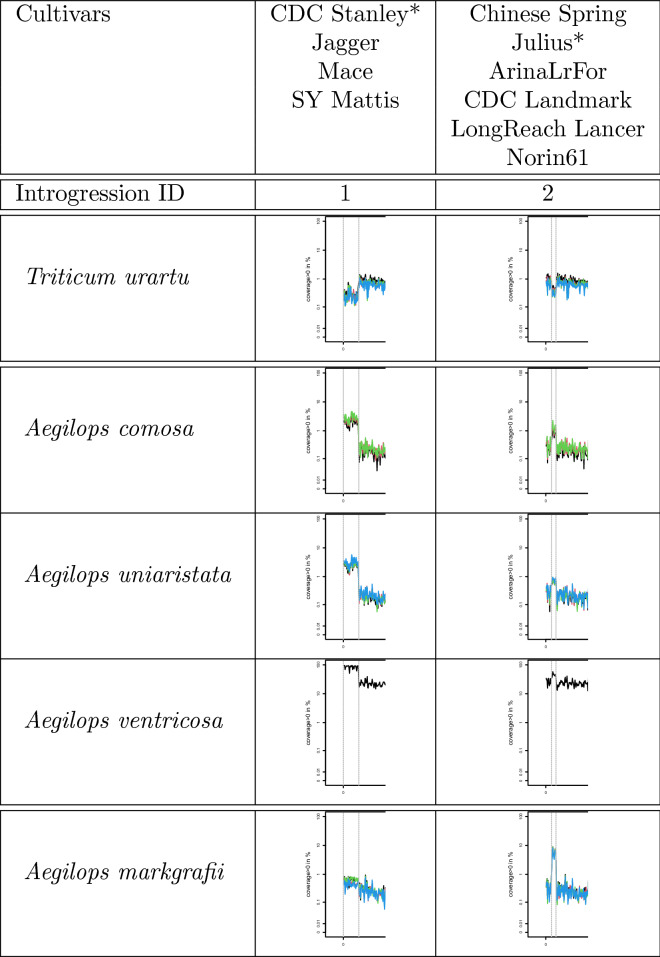
Introgressions on chromosome 2AS (introgressions 1 and 2). Figure shows two clusters of cultivars with two different introgressions, each depicted in one column. In both cases, we detected a region with low coverage of reads from the A-subgenome donor *Triticum urartu* and high coverage of reads from the putative donor species. The cultivars marked with an asterisk were used as the reference genome in the corresponding column.

Thirdly, an introgression on chromosome 2AS (introgression 1) was detected in four cultivars; cv. CDC Stanley, cv. Jagger, cv. Mace, and cv. SY Mattis, with *Aegilops comosa* (2n = 2x = 14, M) and *Aegilops uniaristata* (2n = 2x = 14, N) identified as the putative diploid donors (Fig. 3). It is known from their pedigree that these four cultivars harbour an introgression from tetraploid *Aegilops ventricosa* (2n = 4x = 28, DN)^35–37^, which shares the N subgenome with *Ae. uniaristata*^38^. Using recently published whole genome sequencing (WGS) data for *Ae. ventricosa*^8^, a high-coverage region was detected in the same region on chromosome 2AS (Fig. 3).

In the same region on chromosome 2AS, a second introgression (introgression 2) was detected in the remaining six cultivars (cv. ArinaLrFor, cv. CDC Landmark, cv. Chinese Spring, cv. Julius, cv. LongReach Lancer, and cv. Norin61), with *Aegilops markgrafii* (2n = 2x = 14, C) identified as the putative diploid donor (Fig. 3). Recently, an introgression from *Ae. markgrafii* was produced for this region^39^, but it cannot be the source for these six cultivars because some of them were released a long time ago. Nevertheless, it shows that an introgression from *Ae. markgrafii* is likely in this region.

For chromosome 2D, an introgression (introgression 6) was detected in four cultivars (cv. ArinaLrFor, cv. Jagger, cv. Julius, and cv. SY Mattis) with the putative donor identified as *Ae. markgrafii* or *Ae. umbellulata* (2n = 2x = 14, U). A previously reported introgression in this region^19^ is enriched in elite materials from Western Europe (Lehnert et al*.*, unpublished data) and explains the highest proportion of variance in the grain yield of these materials when cultivated under optimum conditions^40^.

Finally, an introgression on 3DS (introgression 7) from the putative diploid donor species *Ae. comosa* (2n = 2x = 14, M) or *Ae. uniaristata* (2n = 2x = 14, N) was detected in four cultivars (cv. CDC Landmark, cv. CDC Stanley, cv. Julius, and cv. SY Mattis). We suggest that, due to the very narrow native distribution range of these two species, a polyploid taxon such as *Ae. ventricosa* (2x = 4n = 28, DN) or *Ae*. *geniculata* (2x = 4n = 28, MU), which are much more widespread and share a subgenome with these diploid putative donors^38^, is more likely to be the donor of this introgression, similar to the observation for introgression 1. To our knowledge, introgression 7 has not yet been described in the literature.

Most of these introgressions were detected using low cost and low-coverage GBS data^26,27^. To further reduce the costs of genotyping, we tested how much data needs to be generated from CWRs to reasonably describe introgressions in a species. To determine how much data is required to detect an introgression, reads of *Ae. markgrafii* mapped to cv. Julius were exemplarily subsampled from 100 to 1%. Again, the percentage of fixed-size chromosomal windows that was covered by reads was determined (Fig. S2). When using 10% and 1% of the data, higher values were detected for the introgressed region compared with the flanking regions, indicating that even 1% of these low-coverage GBS data is enough to detect introgressions.

Besides the nine introgressions, several additional patterns were detected in the coverage profiles (Supplementary Data 1). These patterns included several chromosomes with increased coverage of reads from *T. boeoticum* and *T. monococcum*, while the coverage profile of the A subgenome donor *T. urartu* was not decreased. Such patterns were detected on chromosome 1AS in cv. ArinaLrFor and cv. Norin61; on chromosome 5AL in cv. ArinaLrFor, cv. CDC Stanley, cv. Jagger, cv. Julius, cv. LongReach Lancer, and cv. SY Mattis; on chromosome 6AS and 6AL in all 10 cultivars; and on chromosome 7AL in cv. CDC Stanley, cv. Mace, and cv. LongReach Lancer (Supplementary Data 1). Previous studies have reported introgressions on wheat chromosomes 5AL and 7AL from *T. monococcum*^41^ and *T. boeoticum*^42^, respectively, although the introgressed regions differ. In contrast, the introgression on chromosome 1AS has been described for cv. Norin61 without any evidence for its donor species or putative origin^43^. Smaller patterns were also detected, for instance, on chromosome 2AL in cv. CDC Stanley and cv. SY Mattis, and on chromosome 5DS in all 10 wheat genomes. These patterns were quite diverse and need to be analysed in more detail in further studies. In the remainder of this manuscript, we focus on the previously uncharacterised introgressions on chromosomes 2AS, 2DL, and 3DS (introgression 2, 6, 7).

### Some introgressions are derived from natural interspecific hybridisations

The observed introgressions were classified into three groups based on their first appearance. The first group (introgressions 3 and 9) consisted of ancient introgressions that probably date back to the time of tetraploidisation of wheat. The second group (introgressions 1, 4, 5, and 8) could be assigned to known interspecific crosses during research and breeding in recent decades. The third group consisted of introgressions whose time of first appearance is still unknown; namely introgression 2 on chromosome 2AS, introgression 6 on chromosome 2DL, and introgression 7 on chromosome 3DS. We checked whether these introgressions were present in some old wheat cultivars that were released before interspecific hybridisation was introduced as a breeding method. For example, the old French wheat cultivar Vilmorin-27, which was released in 1928, is an ancestor of cv. Julius and many contemporary European elite cultivars^32^. Based on publicly available genotyping-by-sequencing (GBS) data for cv. Vilmorin-27^19^, no decreased coverage was detected for the introgressed regions in cv. Julius, indicating that these introgressions were already present in cv. Vilmorin-27 (Fig. S3). To further explore the distribution of these introgressions, the ancestors of cv. Vilmorin-27 were extracted from the pedigree and their seeds were obtained from the genebank at Gatersleben (Table S4). The whole genomes of four plants of each of these cultivars were re-sequenced with low sequencing depth (Table S5) and compared with the cv. Julius reference genome (Fig. S3).

For the low-coverage region on chromosome 2AS (introgression 2), cv. Gros Bleu and cv. Japhet showed normal coverage for most of the region and only low coverage at the end of the region, while the other cultivars showed uniform and high coverage in the complete region, indicating that this introgression was potentially widely utilised in the nineteenth century.

For chromosome 2DL (introgression 6), three different states were observed: First, cv. Hatif Inversable, cv. Dattel, cv. Ble Seigle, and cv. Noe had low coverage in this region, indicating that the analysed individuals did not carry the introgression; second, cv. Japhet had high coverage only at the beginning of the region; and third, cv. Gros Bleu and cv. Bon Fermier had low coverage only at the end of the region. We did not find the complete introgression in any individual of the analysed ancestors.

Three different states were also observed for chromosome 3DS (introgression 7): First, cv. Noe and cv. Dattel had low coverage in this region, indicating that the analysed individuals did not carry the introgression; second, cv. Japhet and cv. Gros Bleu had low coverage only at the very end of this region; and third, cv. Hatif Inversable, cv. Bon Fermier, and cv. Ble Seigle had high coverage in the complete region.

Some of the observed patterns can be traced in the pedigree of cv. Vilmorin-27. For instance, cv. Bon Fermier probably obtained the introgression on chromosome 2DL from cv. Gros Bleu and that on chromosome 3DS from cv. Ble Seigle. Other observed patterns, for example, the introgression on chromosome 2DL in cv. Gros Bleu, could not explained by the pedigree or the current data. Notably, genebank accessions can be heterogeneous, so we may have missed individuals harbouring the complete introgression. Some descendants of cv. Noe may have inherited the complete introgression on chromosome 2DL from cv. Vilmorin-27. Interestingly, cv. Vilmorin-27 is the oldest of the analysed cultivars that carries all three complete introgressions. Nevertheless, single introgressions or parts thereof, as well as combinations, were detected in old cultivars.

### Wide heterogeneity exists within and among genebank accessions

**Figure 4:**
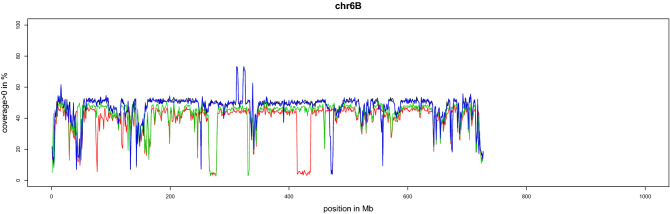
Difference in the coverage profile of chromosome 6B among four individual plants of cv. Krymka. Coverage profiles of the four plants are depicted in four colours (black, blue, red, and green).

To search for the first occurrence of introgressions on chromosome 2DL in wheat, the genomes of old cultivars maintained ex situ were sequenced. To avoid missing introgressions, we did not use materials descended from single seeds in these analyses. Hence, seeds obtained directly from the genebank stocks at Gatersleben were used. The DNA of four individuals per accession was isolated and sequenced (Table S5), and the coverage profiles were compared among the four individuals. We detected remarkable differences in coverage profiles among the four individuals in six of the eight accessions (Supplementary Data 2). Thus, at least one of these four plants carried a chromosomal modification. An extreme example are the three different profiles detected for chromosome 6B in cv. Krymka (shown in red, green, and blue/black in Fig. 4). The differences were large, consisting of several megabases. Interestingly, there were no obvious differences in the phenotypes of these four individual plants when cultivated under greenhouse conditions (Fig. S4 shows the spikes of four individual cv. Krymka plants). We also analysed some CWR accessions using this method. For *Aegilops cylindrica*, one individual from genebank accession AE 656 showed a different coverage profile for chromosomes 1D, 3D, 4D, and 5D, indicating that it also carries introgressions (Fig. S5). This is plausible given earlier studies on the potential for gene transfer in *Ae. cylindrica*^44^.

### Introgressed regions harbour homologues of resistance genes

Introgressions have often been initiated to transfer resistance genes from CWRs into breeding materials. Often, the resistance gene is then tracked with linked markers in the introgression or in its flanking sequences^45^. We utilised recently published markers to identify the candidate gene *PGSB_gene_1945*^35^, which may be homologous to the leaf rust resistance gene *LrM*^39^. In addition, we used the recently described yellow rust resistance genes *Yr5* and *Yr7*^46^ for homology-based gene prediction within the described introgression regions in the 10 RQAs. Besides a high amino acid sequency identity in a pairwise alignment of predicted and reference proteins (≥ 80%), all predicted genes had the same number of coding exons (ce) as the reference gene (rce) and encoded a protein of similar length (aa vs. raa) (Table S6).

For *PGSB_gene_1945*, homology-based gene prediction using GeMoMa predicted 10 genes—one in each RQA on chromosome 2AS located in the region of the introgression. Interestingly, there were only two sequences of these predicted genes. For cv. CDC Stanley, cv. Jagger, cv. Mace, and cv. SY Mattis, the predicted gene product showed 100% amino acid sequence identity to the product of *PGSB_gene_1945*. The other six cultivars harboured a gene whose predicted product showed 95.6% amino acid sequence identity to the product of *PGSB_gene_1945*. These two groups of cultivars were completely consistent with the clusters formed based on the introgression on chromosome 2AS.

Two putative homologues of the resistance gene *Yr7* were detected on chromosome 2B. The homologue in this region in cv. CDC Landmark, cv. CDC Stanley, and cv. Mace, encoded a protein with 99.9% amino acid sequence identity to Yr7. The pedigree of these three cultivars indicates that *Yr7* was introduced from the tetraploid durum wheat cv. Iumillo into the wheat cv. Thatcher^47^. The homologue in this region in cv. CDC Landmark, cv. CDC Stanley, cv. Mace, and cv. SY Mattis encoded a protein with 84.9% amino acid sequence identity to Yr7. The remaining three cultivars did not harbour any genes encoding a protein with at least 80% amino acid sequence identity to Yr7.

Three homologues of the resistance gene *Yr5* were identified. The *Yr5* homologue on chromosome 2B in cv. Julius and cv. LongReach Lancer encoded a protein with 91.1% amino acid sequence identity to Yr5. This predicted gene was located within a potential introgression from *T. timopheevii*, which is present on chromosome 2BL in both cultivars. The second and third predicted genes were located within the described introgression on chromosome 2DL. The homologue in cv. Jagger and cv. Julius encoded a protein with 99.2% amino acid sequence identity to Yr5, and that in cv. ArinaLrFor and cv. SY Mattis encoded a protein with 98.1% amino acid sequence identity to Yr5. Interestingly, the predicted genes in cv. Jagger and cv. Julius were identical to the *Yr5* allele from cv. Claire^46^. Since the predicted genes were located within the introgression on chromosome 2DL, we speculate that there have been at least two independent events or mutations at this locus. The predicted genes were located in the first part of the introgression that was present in cv. Japhet, cv. Gros Bleu and cv. Bon Fermier. Hence, these cultivars might harbour an allele conferring increased resistance against yellow rust.

## Discussion

Hybridisation between wheat and its wild relatives occurs naturally, but can also be conducted during breeding to introduce beneficial traits into elite wheat breeding material. Here, we have shown for the first time that introgressions and their putative donor species can be identified without prior knowledge of the pedigree. Moreover, these introgressions can be easily traced, facilitating the desirable but challenging task to decrease introgression fragments to reduce linkage drag. The described bioinformatics method can be used for GBS, whole genome exome capture, and whole genome resequencing data. Large introgressions can be detected from GBS data, which is relatively cheap to obtain. Smaller introgressions may be identified from data generated using more expensive, but also more informative techniques such as exome capture and whole genome resequencing.

We have identified several known and previously unknown introgressions in 10 RQAs of wheat. Some of these introgressions are well described and were conducted in the framework of research and breeding programs. Interspecies crosses in wheat were described for the first time in the last quarter of the nineteenth century^48^. However, we also identified introgressions in old cultivars released in the first half of the nineteenth century. This observation is consistent with findings of introgressions from *T. monococcum* in bread wheat cv. Mediterranean^41^ released in 1837. We hypothesise that the introgressions found in old cultivars resulted from spontaneous natural interspecific crosses that were subsequently selected. For various reasons, it is almost impossible to determine the exact origin of these introgressions. These reasons include (i) the common subgenomes of wheat relatives; (ii) the heterogeneity of old landraces; (iii) conservation issues over the centuries, including seed exchange and multiplication leading to the potential loss of alleles and/or contamination that have affected the integrity of genebank accessions; and (iv) the lack of data on the exact timing and location of the first occurrence of these introgressions. Nevertheless, we were able to narrow down the putative donors for several introgressions. These findings will be useful in the search for alternative alleles in donor species that might be valuable for crop improvement under changing climatic conditions and increasingly severe biotic stress.

Further, within the introgressed DNA regions, we identified genes showing strong sequence identity to known resistance genes. Further research is required to test their efficacy against pathogens. Interestingly, the old cv. Noe without the 2DL introgression was described as quite susceptible to rust. In contrast, cv. Gros Bleu and cv. Japhet, which are different selections of cv. Noe that carry a large and a small fragment of the 2DL introgression, respectively, are much more resistant to rust than is cv. Noe^49^. Within the common part of this introgression, we identified a homologue of the resistance gene *Yr5*. The discovery of a *Yr5* homologue in the introgressed region on chromosome 2DL of cv. Julius and cv. Jagger proves that the *Yr5* allele of cv. Claire is indeed located on chromosome 2D and not on chromosome 2B like the original *Yr5*^46^. In addition, we predicted another allele of *Yr5* in the introgressed region on chromosome 2DL of cv. ArinaLrFor and cv. SY Mattis, indicating that there may have been independent introgression events. We also detected *Yr5* orthologs on chromosome 2BL and 2DL in cv. Julius, demonstrating that the combination of introgressions on different chromosomes allows for stacking of homoeologous resistance genes. Finding homologue resistance genes in CWRs that can be introgressed into different subgenomes of wheat might facilitate combining resistance genes to yield durable and broadly resistant lines.

Interestingly, we detected *Yr5* at the beginning of the introgression on chromosome 2DL. Some wheat cultivars, e.g. cv. Japhet., carry only the first part of the introgression. Nevertheless, the complete introgression on chromosome 2DL is highly enriched in Western European elite winter wheat materials, indicating that at least one other trait may be affected by this introgression, potentially by a gene or genes located in the latter part.

Genome assemblies have been published for some wheat relatives^50–55^, but not for all of them. Therefore, for building a wheat super-pangenome, it is more promising to conduct genome sequencing and assembly for CWRs than for elite cultivars that share a large part of the genome with already sequenced wheat cultivars^56^. With the genome assemblies of wheat CWRs and the described coverage method, large collections of wheat materials could be analysed to detect natural or induced introgressions, for example, in genebank accessions or breeding material.

Genebank accessions are heterogeneous to some extent, despite careful management including splitting of phenotypically different plants within an accession^57–59^. For this reason, introgression events and important alleles may be lost when using bulks or materials descended from a single seed^60^. Therefore, it needs to be discussed whether original genebank accessions should be split into independent lines on the basis of genetic data. It has been proposed that duplicates of genebank accessions should be removed to save money and reduce the time and materials needed for their management, while preserving genetic diversity^61^. We suggest that duplicates should not be identified by detecting SNPs in one seed per accession, but on the basis of a combination of SNPs and coverage analysis for multiple seeds per accession. The capacity gained by removing true duplicates could be used for splitting and maintaining additional genebank accessions.

Genomic data for entire genebank collections (https://www.pflanzenforschung.de/de/forschung-plant-2030/projekte/274/detail#english) will be soon be available for analysis. This could accelerate the breeding process, if accessions of crop species harbouring interesting introgressions could be identified and used immediately. Although we demonstrate the utility of the method for wheat, it could easily be applied to other species.

## Methods

### Plant materials and whole genome resequencing

For each selected genebank accession (Table S4), 10 seeds were retrieved from the Federal ex situ Genebank for Agricultural and Horticultural Plants of Germany maintained at the Leibniz Institute of Plant Genetics and Crop Plant Research (IPK) in Gatersleben. Seeds were sown in small pots (Ø 5 cm) filled with a soil mixture of 70% Substrat 1 (Fa. Klasmann-Deilmann, Geeste, Germany), 20% compost, and 10% sand. Seedlings were grown under controlled greenhouse conditions (14-h/10-h day/night, ~ 15–18/ ~ 12–15 °C day/night). For DNA extraction, fresh leaves were cut from four plants of each accession at the two-leaf stage. The leaves were dried with silica gel at room temperature for 10 days. At the three-leaf stage, plants were transferred to pots (Ø 14 cm) filled with a soil mixture of 40% Substrat 2 (Fa. Klasmann-Deilmann), 50% compost, and 10% sand, and grown under controlled greenhouse conditions (16-h/8-h day/night, ~ 20–23/ ~ 17–20 °C day/night) until maturity.

Total DNA was extracted separately from dried leaf tissue of four individuals per genebank accession using a DNeasy Plant Kit (QIAGEN, Hilden, Germany) according to the manufacturer’s instructions. The WGS library (Nextera DNA Flex, genomic DNA input: 100–500 ng) was prepared according to the standard protocols of the manufacturer (Illumina, Inc., San Diego, CA, USA). The library was quantified by qPCR (KAPA Library Quantification Kit; KAPA Biosystems, Wilmington, MA, USA) and sequenced on the NovaSeq 6000 platform (Illumina, Inc.; run type: SP PE 151) at the IPK.

### Coverage analysis

Raw sequencing data were adapter- and quality-trimmed with Trim Galore (version 0.4.0; non default parameters: quality ≥ 30, read length ≥ 50; https://github.com/FelixKrueger/TrimGalore). Trimmed reads were individually mapped against the wheat RQAs using BWA-mem (v0.7.15-r1140) (Li, 2013). Unmapped reads, supplementary reads, and non-primary alignments were removed from mapped reads using SAMtools (–F 2308) before computing depth. Finally, depth was aggregated and visualised with R^62^. For visualisation, coverage was displayed on a logarithmic scale using log(x + ε) transformation with ε = 0.01.

### Gene predictions

For predicting resistance genes in introgressed regions, all 10 wheat RQAs^63,64^ were analysed using GeMoMa (version 1.8), a homology-based gene prediction program that allows annotations to be transferred from one genome to another. Known resistance genes located in the potential introgressions were used as reference genes. We used the known resistance genes *Yr5* and *Yr7*^46^, as well as TraesCS2A01G040000, which was identified in Chinese Spring using the primers AX_948171722AS and AX_945219402AS^39^, and corresponds to *PGSB_gene_1945* in the cv. Jagger RQA^35^. Predicted genes were filtered based on the amino acid identity of their encoded products to the reference protein (≥ 80%).

### Statement

We confirm that experimental research and field studies on plants (either cultivated or wild), including the collection of plant material, comply with relevant institutional, national, and international guidelines and legislation.

## Supplementary Information

Supplementary Information 1.

Supplementary Information 2.

Supplementary Figures.

Supplementary Tables.

## Data Availability

Publicly available data were downloaded from^65^ https://doi.org/10.5447/IPK/2019/18. Additional genomic data was downloaded from EMBL ENA with the following IDs: ERR2936519 and ERR2936122 (GBS data of cv. Vilmorin-27), SRR2061020 (*Th. ponticum*), SRR13484813 (WGS data of *Ae. ventricosa*), and PRJNA601245 (GBS data of *Triticum* and *Ae. triuncialis*). Own sequence data is available from ENA (EMBL-EBI) with the following ID PRJEB49121. Please note that several classification schemes have been proposed for wheat and *Aegilops*, e.g., based on morphological, cytogenetic, and genomic characteristics. Sometimes the published information is incorrect or misleading. In this study, we consider the taxon names used by Sharma et al.^65^. However, we cannot change existing database entries. Overviews of most important wheat and *Aegilops* classifications can be found in recently published literature^65^.
