# Supplementary material for: Detecting major introgressions in wheat and their putative origins using coverage analysis
Source: Sci Rep. 2022 Feb 3;12:1908. doi: 10.1038/s41598-022-05865-w (PMC8813953; doi:10.1038/s41598-022-05865-w)

Summary of coverage analysis for all 10 wheat reference quality assemblies (RQAs). File is a large PDF document, where each page contains all information for one chromosome of wheat. Columns correspond to wheat RQAs and rows correspond to CWR species. Each plot shows coverage data from multiple accessions of a CWR displayed in different colours.

Chromosome 1A

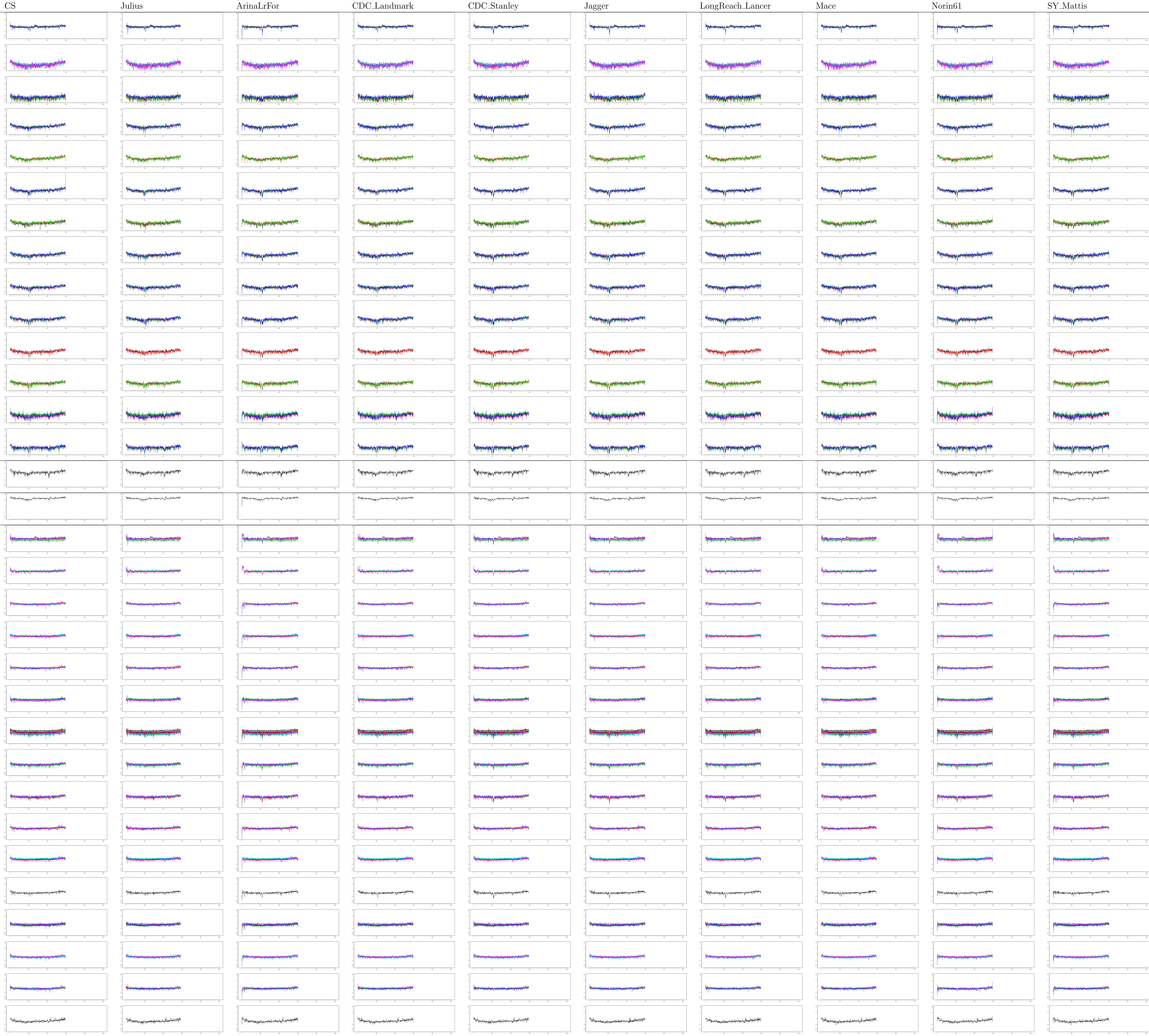

Genome	Species
A	*Triticum urartu*
S	*Aegilops speltoides*
D	*Aegilops tauschii*
C	*Aegilops markgrafii*
U	*Aegilops umbellulata*
T	*Aegilops mutica*
M	*Aegilops comosa*
N	*Aegilops uniaristata*
S^l	*Aegilops longissima*
S^s	*Aegilops searsii*
S^{sh}	*Aegilops sharonensis*
S^b	*Aegilops bicornis*
V	*Dasypyrum villosum*
Ta	*Taeniatherum caput medusae*
$EEEE^{st}E^{st}$	*Thinopyrum ponticum*
DN	*Aegilops ventricosa*
A^b	*Triticum boeoticum*
A^b	*Triticum monococcum*
BA	*Triticum carthlicum*
BA	*Triticum dicoccoides*
BA	*Triticum dicoccon*
BA	*Triticum polonicum*
BA	*Triticum durum*
BA	*Triticum turanicum*
BA	*Triticum turgidum*
GA^t	*Triticum armeniacum*
GA^t	*Triticum timopheevii*
BA^tA^b	*Triticum zhukovskyi*
BAD	*Triticum spelta*
BAD	*Triticum sphaerococcum*
BAD	*Triticum compactum*
UC	*Aegilops triuncialis*

Chromosome 1B

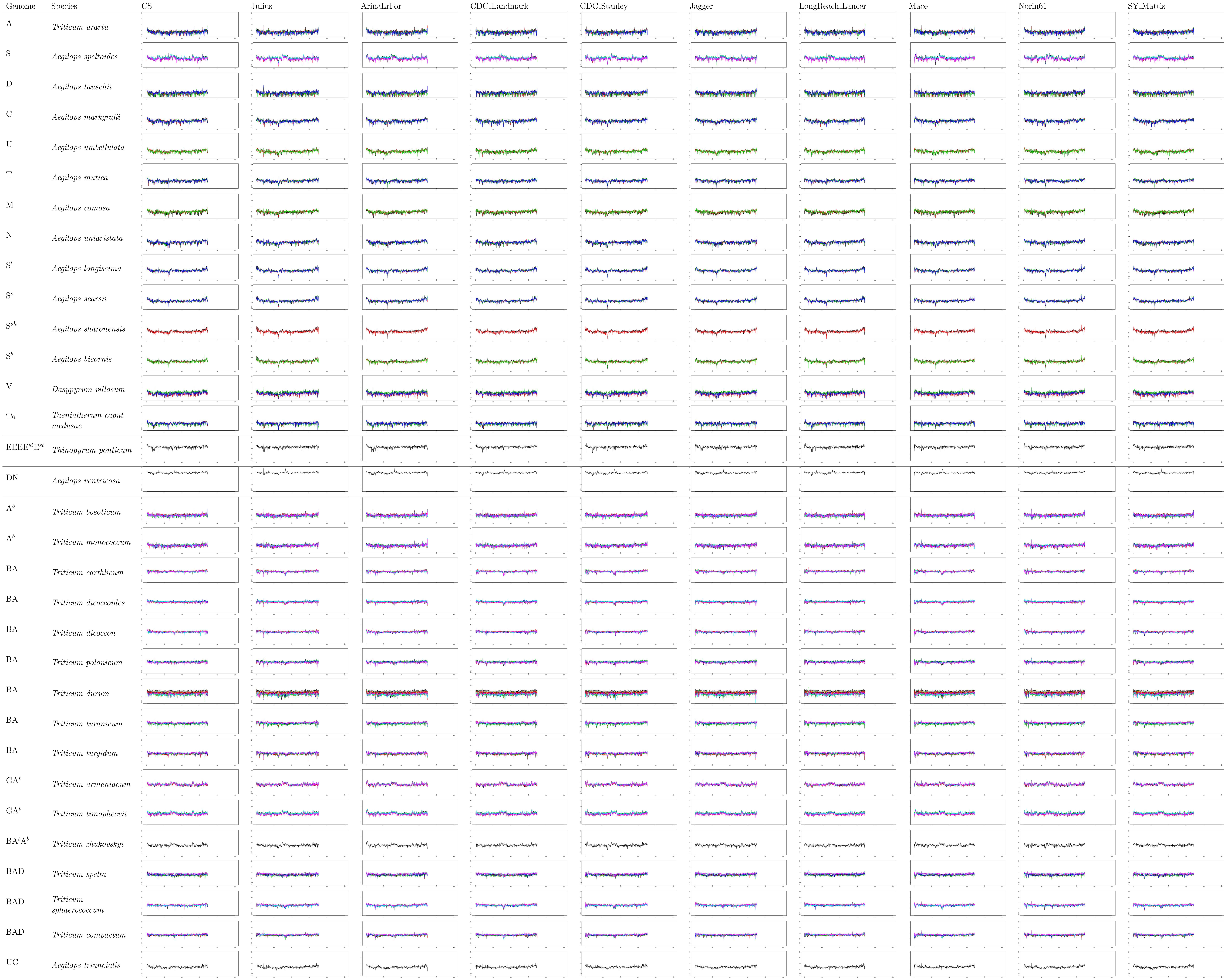

Chromosome 1D

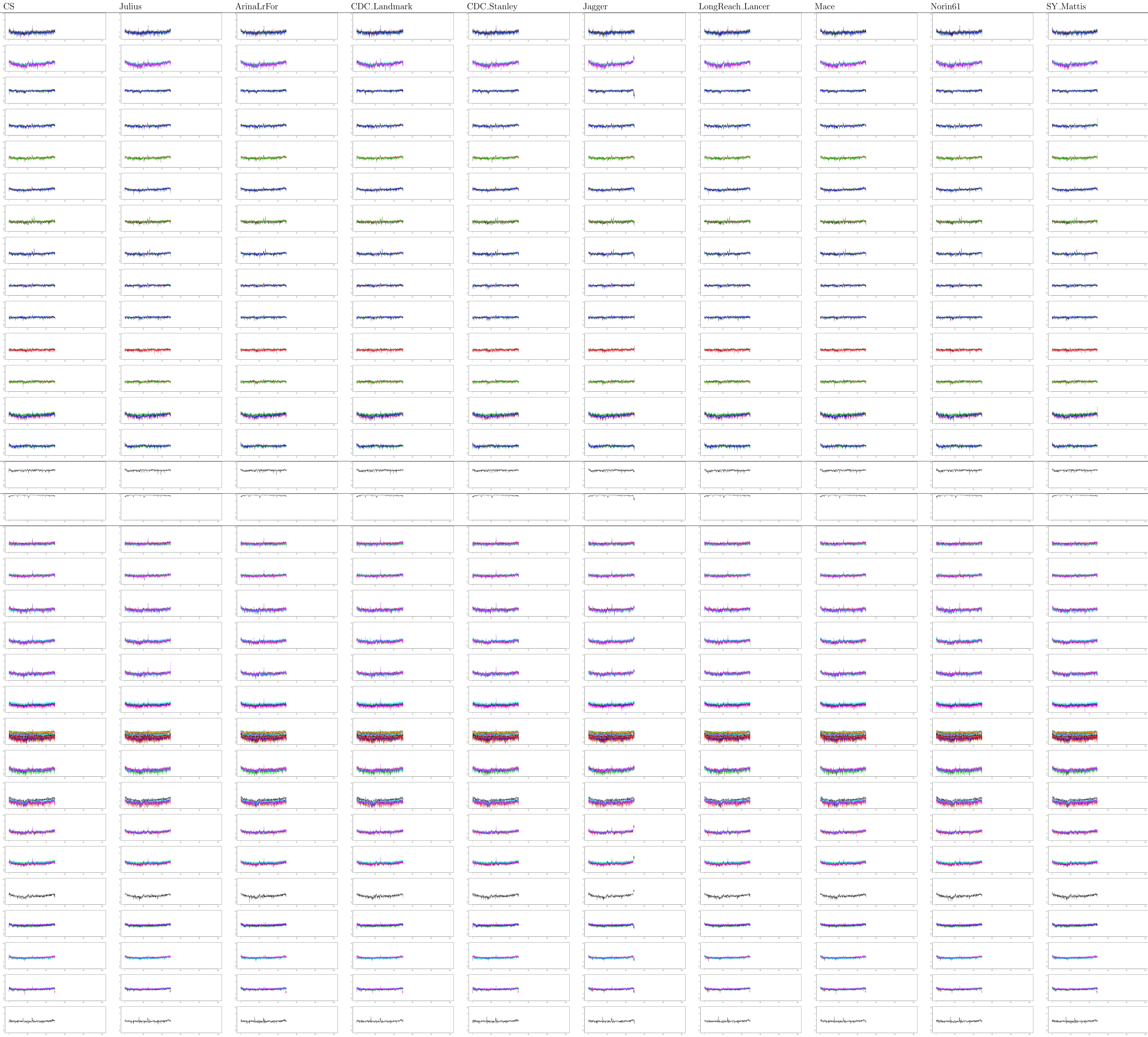

Genome	Species
A	*Triticum urartu*
S	*Aegilops speltoides*
D	*Aegilops tauschii*
C	*Aegilops markgrafii*
U	*Aegilops umbellulata*
T	*Aegilops mutica*
M	*Aegilops comosa*
N	*Aegilops uniaristata*
S^l	*Aegilops longissima*
S^s	*Aegilops searsii*
S^{sh}	*Aegilops sharonensis*
S^b	*Aegilops bicornis*
V	*Dasypyrum villosum*
Ta	*Taeniatherum caput medusae*
$EEEE^{st}E^{st}$	*Thinopyrum ponticum*
DN	*Aegilops ventricosa*
A^b	*Triticum boeoticum*
A^b	*Triticum monococcum*
BA	*Triticum carthlicum*
BA	*Triticum dicoccoides*
BA	*Triticum dicoccon*
BA	*Triticum polonicum*
BA	*Triticum durum*
BA	*Triticum turanicum*
BA	*Triticum turgidum*
GA^t	*Triticum armeniacum*
GA^t	*Triticum timopheevii*
BA^tA^b	*Triticum zhukovskyi*
BAD	*Triticum spelta*
BAD	*Triticum sphaerococcum*
BAD	*Triticum compactum*
UC	*Aegilops triuncialis*

Chromosome 2A

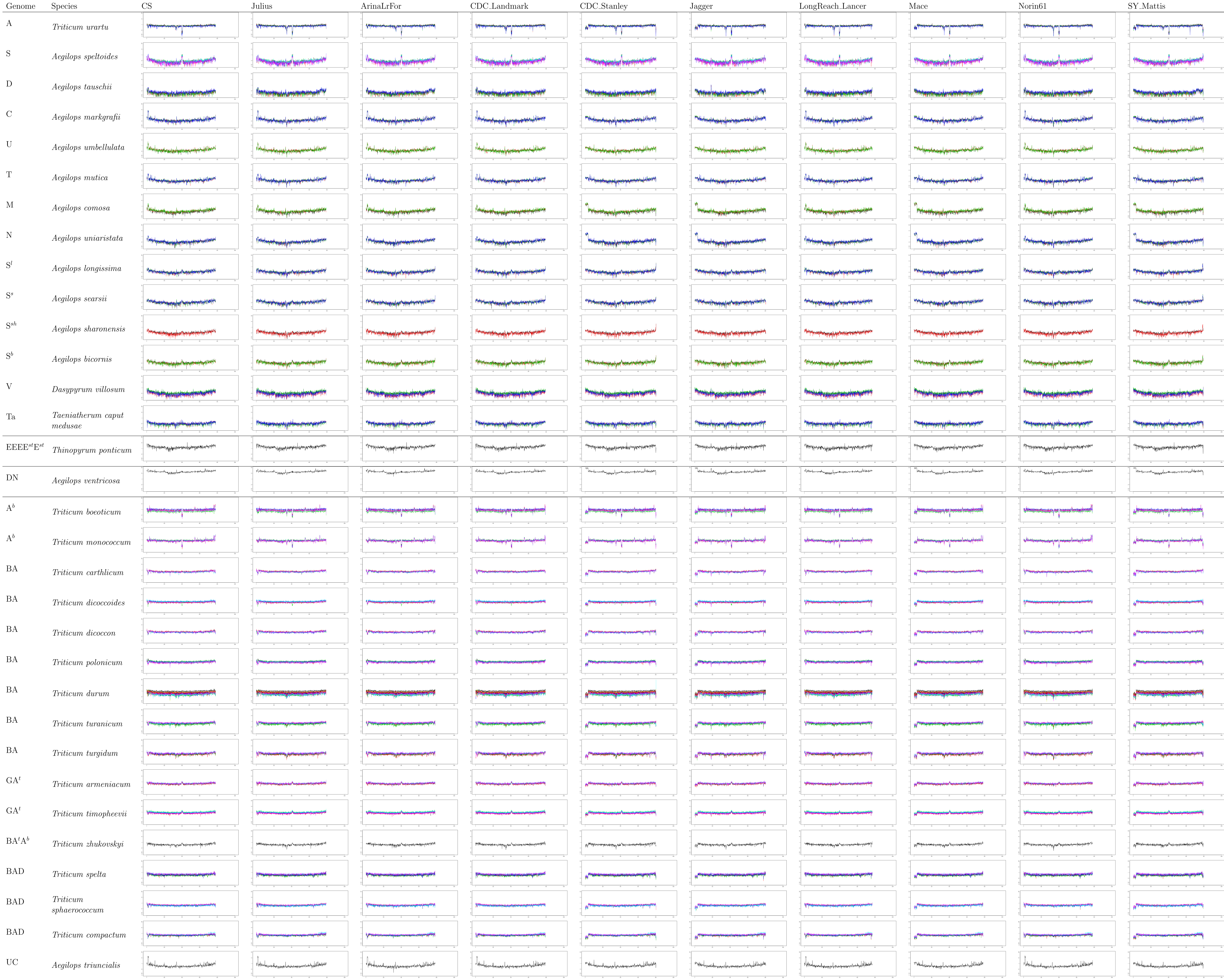

Chromosome 2B

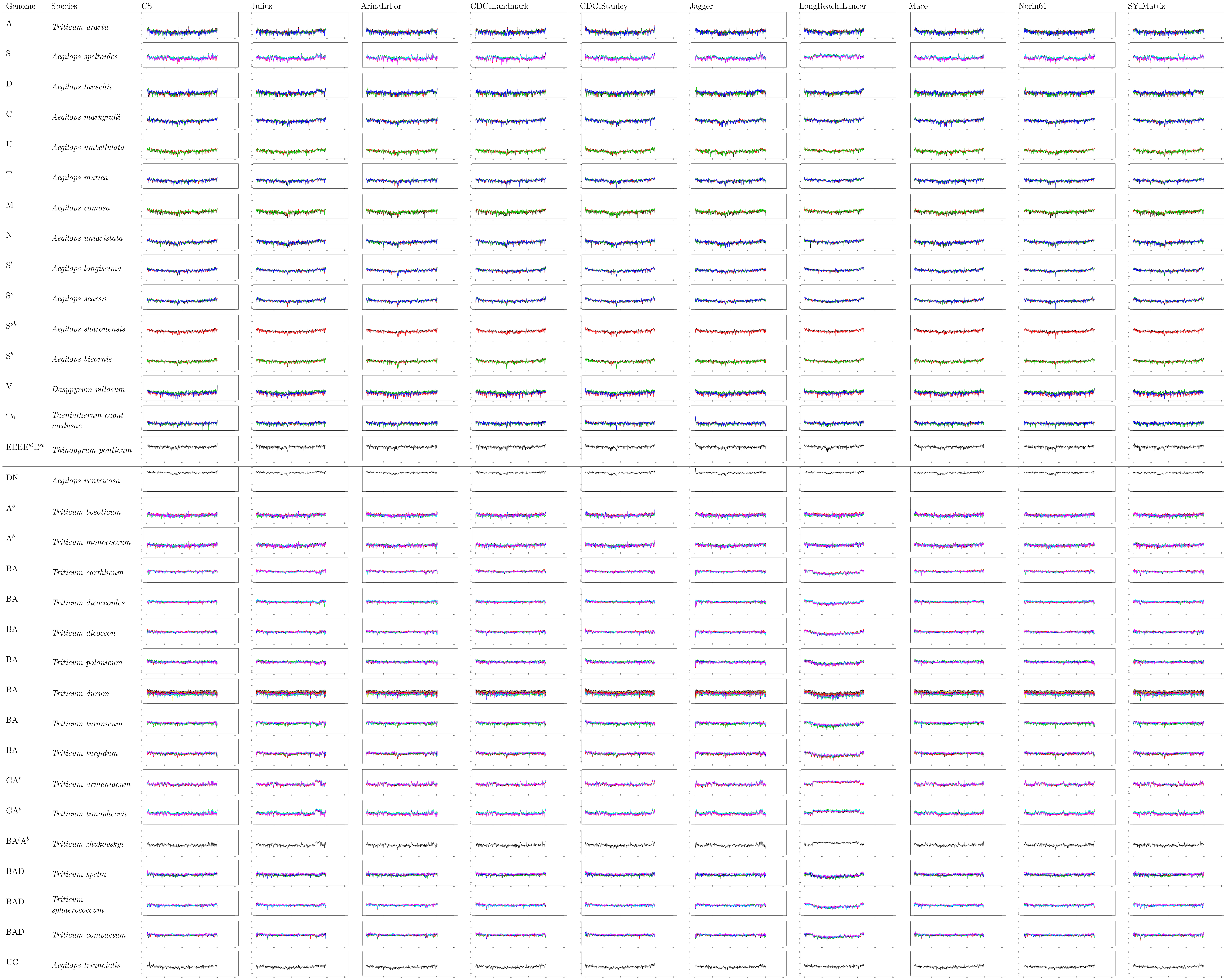

Chromosome 2D

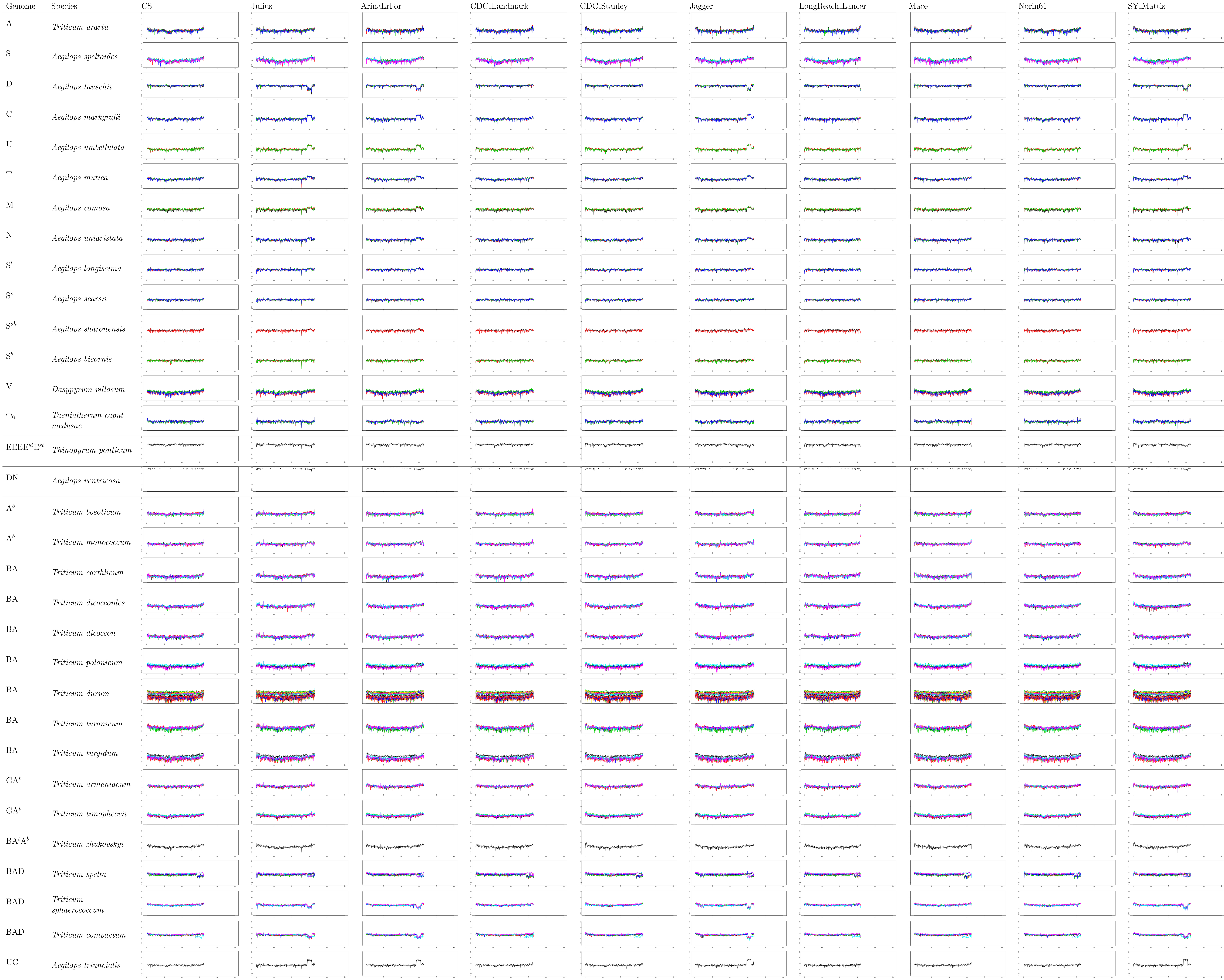

Chromosome 3A

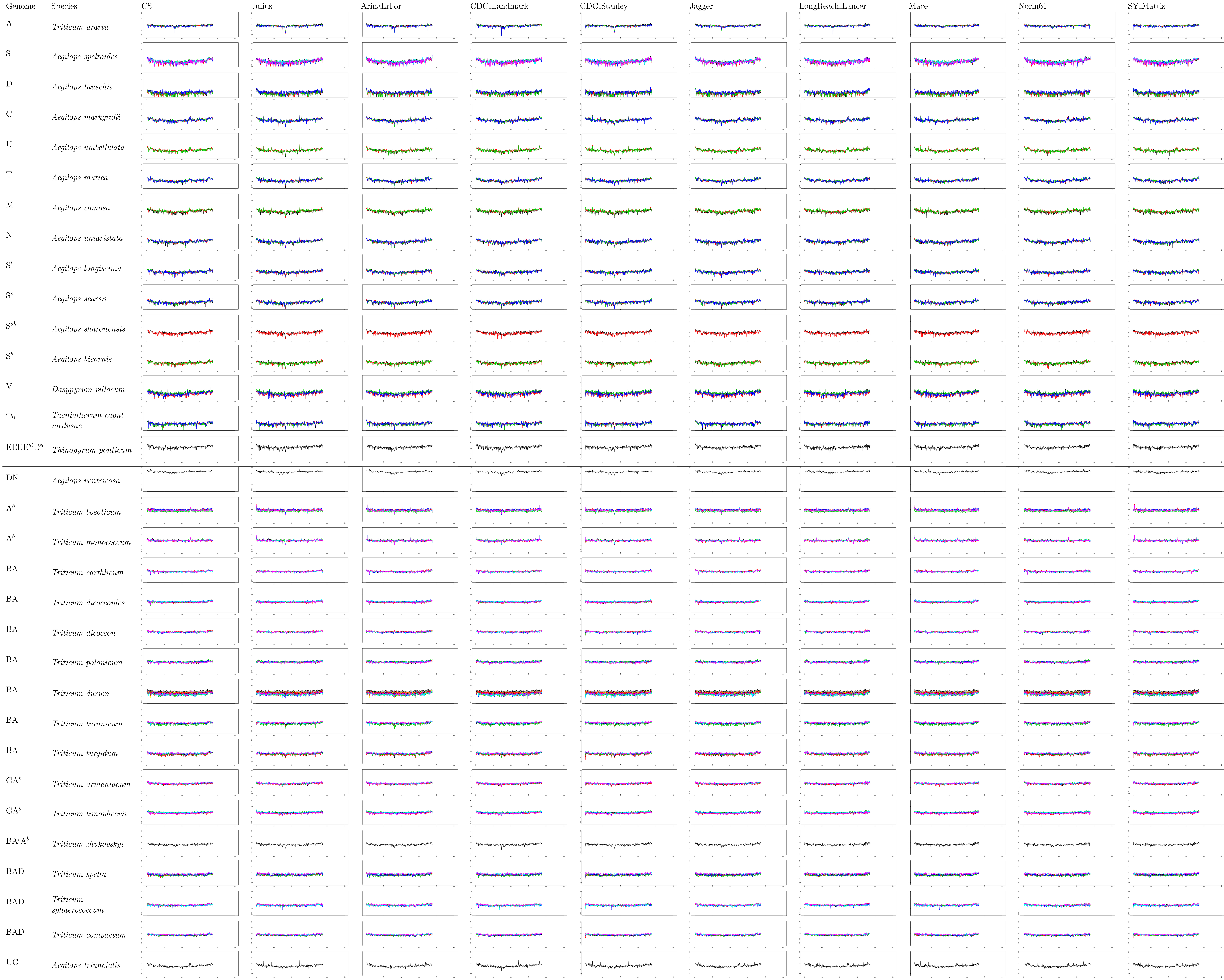

Chromosome 3B

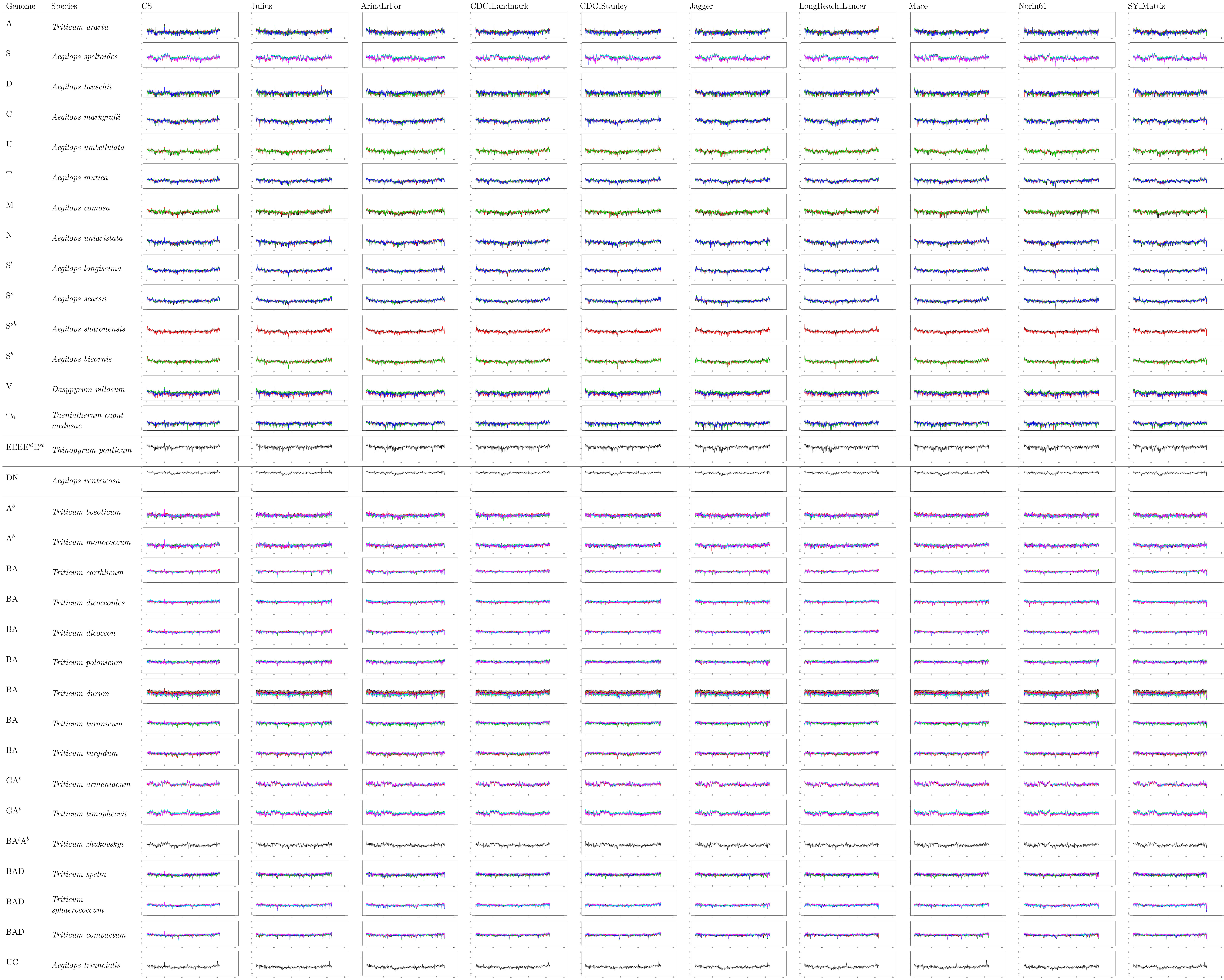

Chromosome 3D

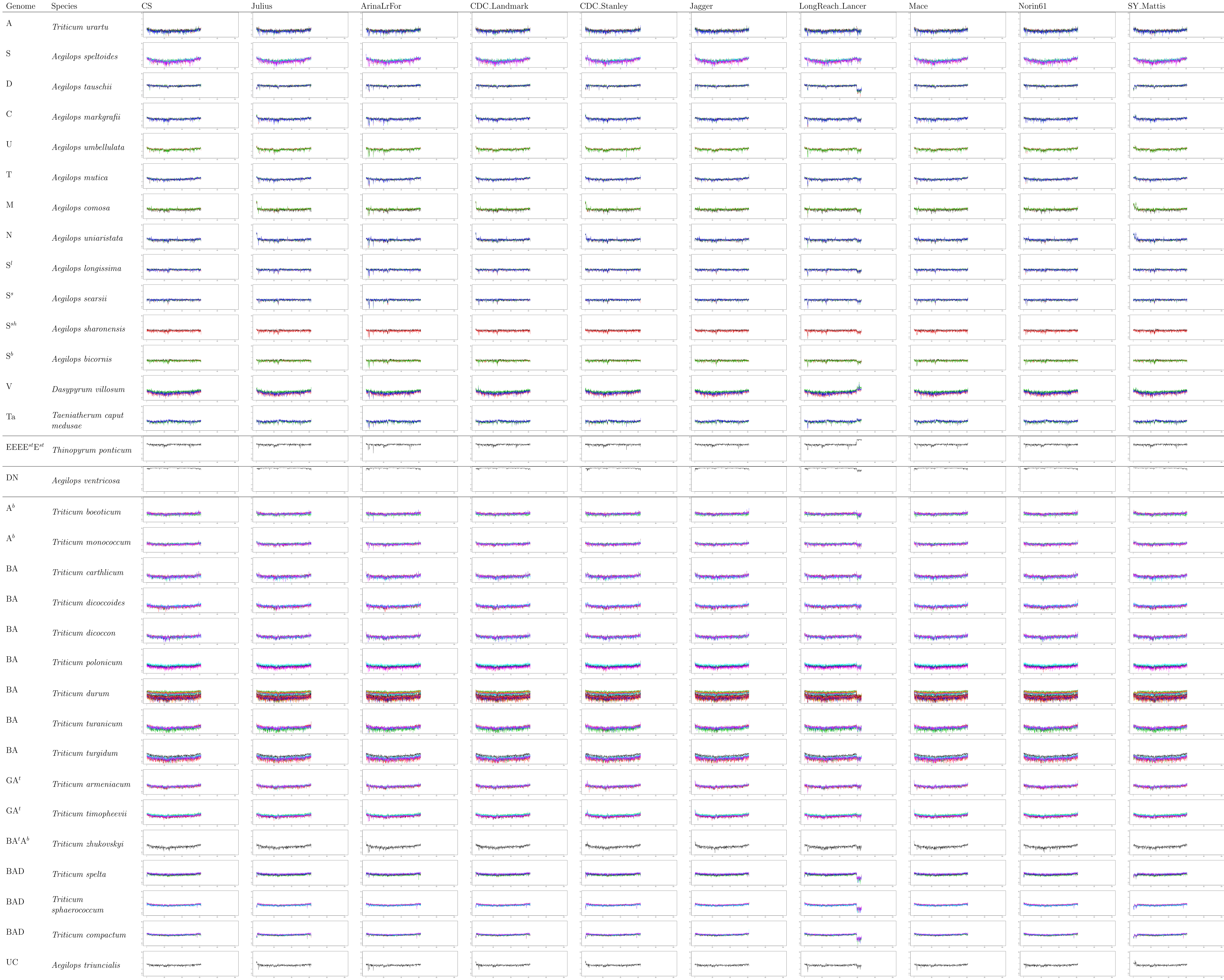

Chromosome 4A

Genome	Species
A	*Triticum urartu*
S	*Aegilops speltoides*
D	*Aegilops tauschii*
C	*Aegilops markgrafii*
U	*Aegilops umbellulata*
T	*Aegilops mutica*
M	*Aegilops comosa*
N	*Aegilops uniaristata*
S^l	*Aegilops longissima*
S^s	*Aegilops searsii*
S^{sh}	*Aegilops sharonensis*
S^b	*Aegilops bicornis*
V	*Dasypyrum villosum*
Ta	*Taeniatherum caput medusae*
$EEEE^{st}E^{st}$	*Thinopyrum ponticum*
DN	*Aegilops ventricosa*
A^b	*Triticum boeoticum*
A^b	*Triticum monococcum*
BA	*Triticum carthlicum*
BA	*Triticum dicoccoides*
BA	*Triticum dicoccon*
BA	*Triticum polonicum*
BA	*Triticum durum*
BA	*Triticum turanicum*
BA	*Triticum turgidum*
GA^t	*Triticum armeniacum*
GA^t	*Triticum timopheevii*
BA^tA^b	*Triticum zhukovskyi*
BAD	*Triticum spelta*
BAD	*Triticum sphaerococcum*
BAD	*Triticum compactum*
UC	*Aegilops triuncialis*

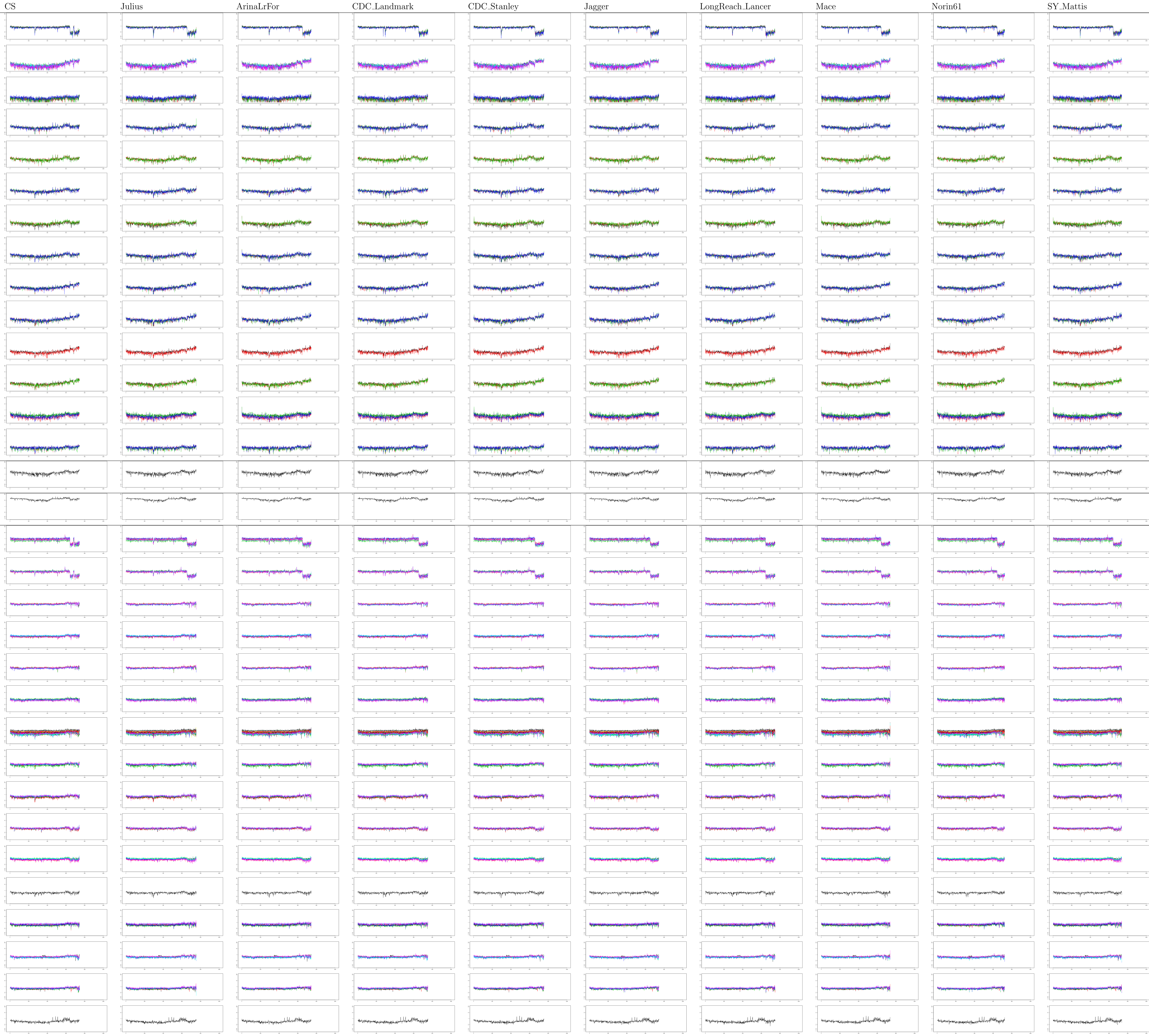

Chromosome 4B

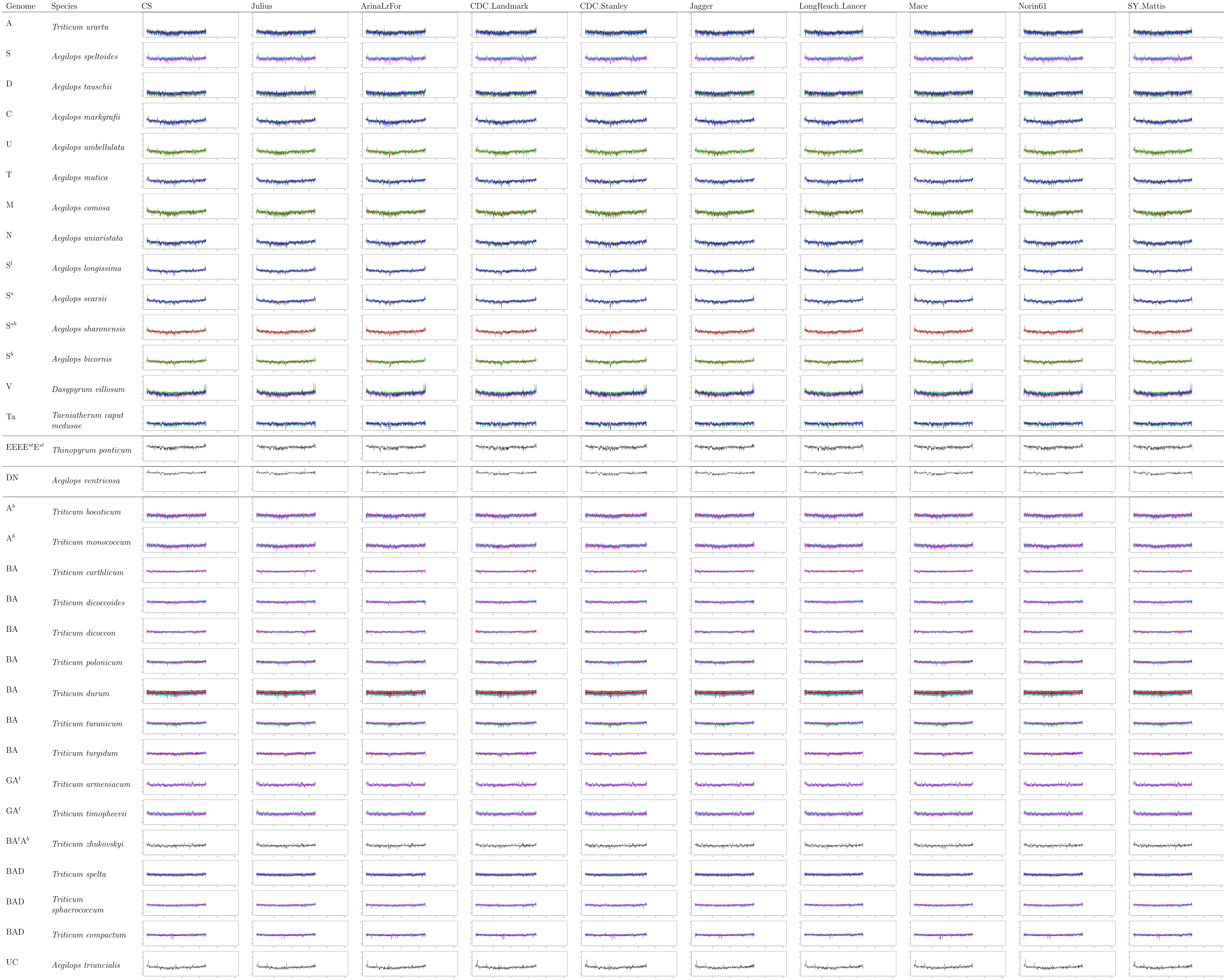

Chromosome 4D

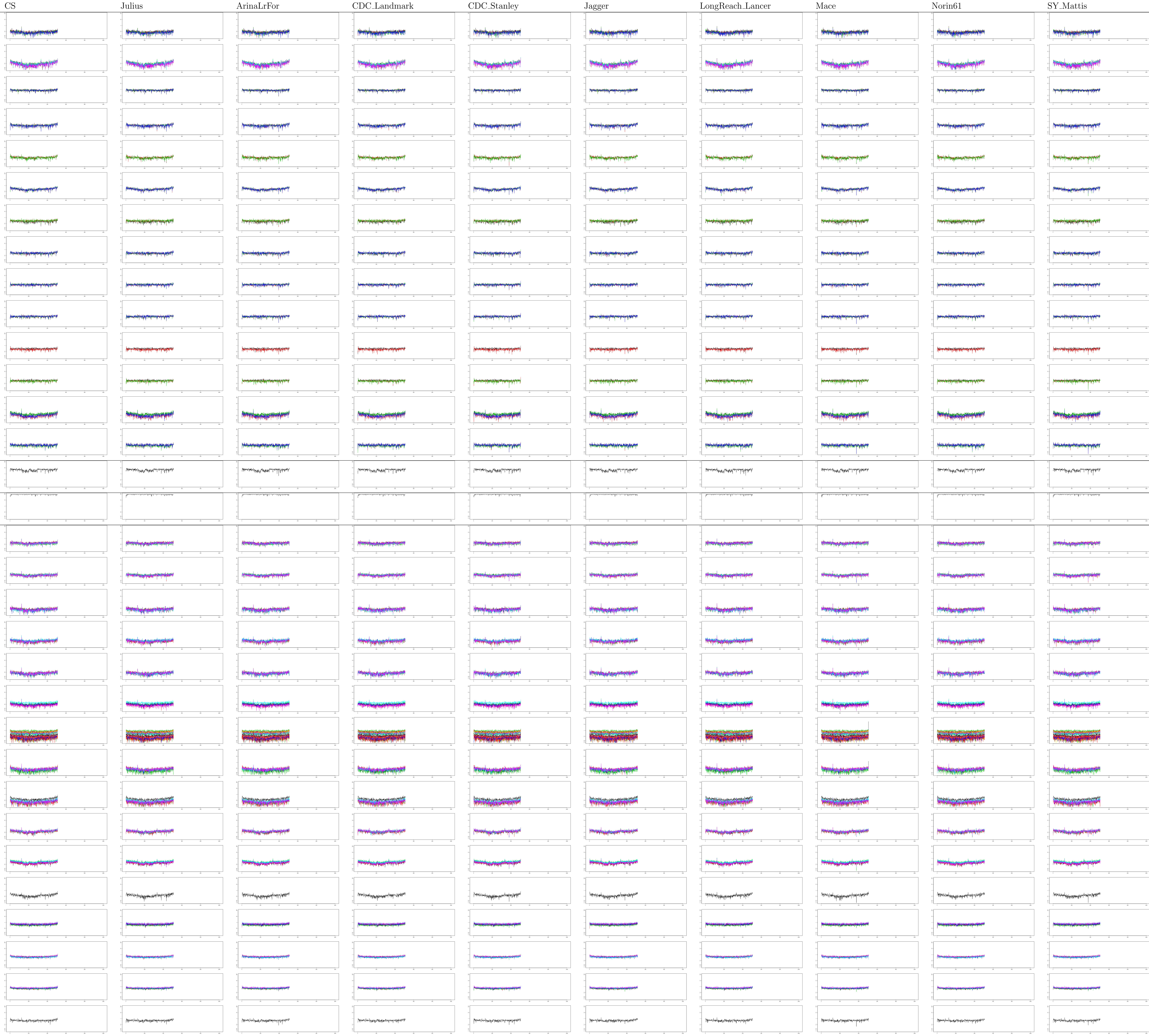

Genome	Species	CS	Julius	ArinaLrFor	CDC_Landmark	CDC_Stanley	Jagger	LongReach_Lancer	Mace	Norin61	SY_Mattis
A	*Triticum urartu*										
S	*Aegilops speltoides*										
D	*Aegilops tauschii*										
C	*Aegilops markgrafii*										
U	*Aegilops umbellulata*										
T	*Aegilops mutica*										
M	*Aegilops comosa*										
N	*Aegilops uniaristata*										
S^l	*Aegilops longissima*										
S^s	*Aegilops searsii*										
S^{sh}	*Aegilops sharonensis*										
S^b	*Aegilops bicornis*										
V	*Dasypyrum villosum*										
Ta	*Taeniatherum caput medusae*										
$EEEE^{st}E^{st}$	*Thinopyrum ponticum*										
DN	*Aegilops ventricosa*										
A^b	*Triticum boeoticum*										
A^b	*Triticum monococcum*										
BA	*Triticum carthlicum*										
BA	*Triticum dicoccoides*										
BA	*Triticum dicoccon*										
BA	*Triticum polonicum*										
BA	*Triticum durum*										
BA	*Triticum turanicum*										
BA	*Triticum turgidum*										
GA^t	*Triticum armeniacum*										
GA^t	*Triticum timopheevii*										
BA^tA^b	*Triticum zhukovskyi*										
BAD	*Triticum spelta*										
BAD	*Triticum sphaerococcum*										
BAD	*Triticum compactum*										
UC	*Aegilops triuncialis*										

Chromosome 5A

Genome	Species	CS	Julius	ArinaLrFor	CDC_Landmark	CDC_Stanley	Jagger	LongReach_Lancer	Mace	Norin61	SY_Mattis
A	*Triticum urartu*										
S	*Aegilops speltoides*										
D	*Aegilops tauschii*										
C	*Aegilops markgrafii*										
U	*Aegilops umbellulata*										
T	*Aegilops mutica*										
M	*Aegilops comosa*										
N	*Aegilops uniaristata*										
S^{l}	*Aegilops longissima*										
S^{s}	*Aegilops searsii*										
S^{sh}	*Aegilops sharonensis*										
S^{b}	*Aegilops bicornis*										
V	*Dasypyrum villosum*										
Ta	*Taeniatherum caput medusae*										
$EEEE^{st}E^{st}$	*Thinopyrum ponticum*										
DN	*Aegilops ventricosa*										
A^{b}	*Triticum boeoticum*										
A^{b}	*Triticum monococcum*										
BA	*Triticum carthlicum*										
BA	*Triticum dicoccoides*										
BA	*Triticum dicoccon*										
BA	*Triticum polonicum*										
BA	*Triticum durum*										
BA	*Triticum turanicum*										
BA	*Triticum turgidum*										
GA^{t}	*Triticum armeniacum*										
GA^{t}	*Triticum timopheevii*										
$BA^{t}A^{b}$	*Triticum zhukovskyi*										
BAD	*Triticum spelta*										
BAD	*Triticum sphaerococcum*										
BAD	*Triticum compactum*										
UC	*Aegilops triuncialis*										

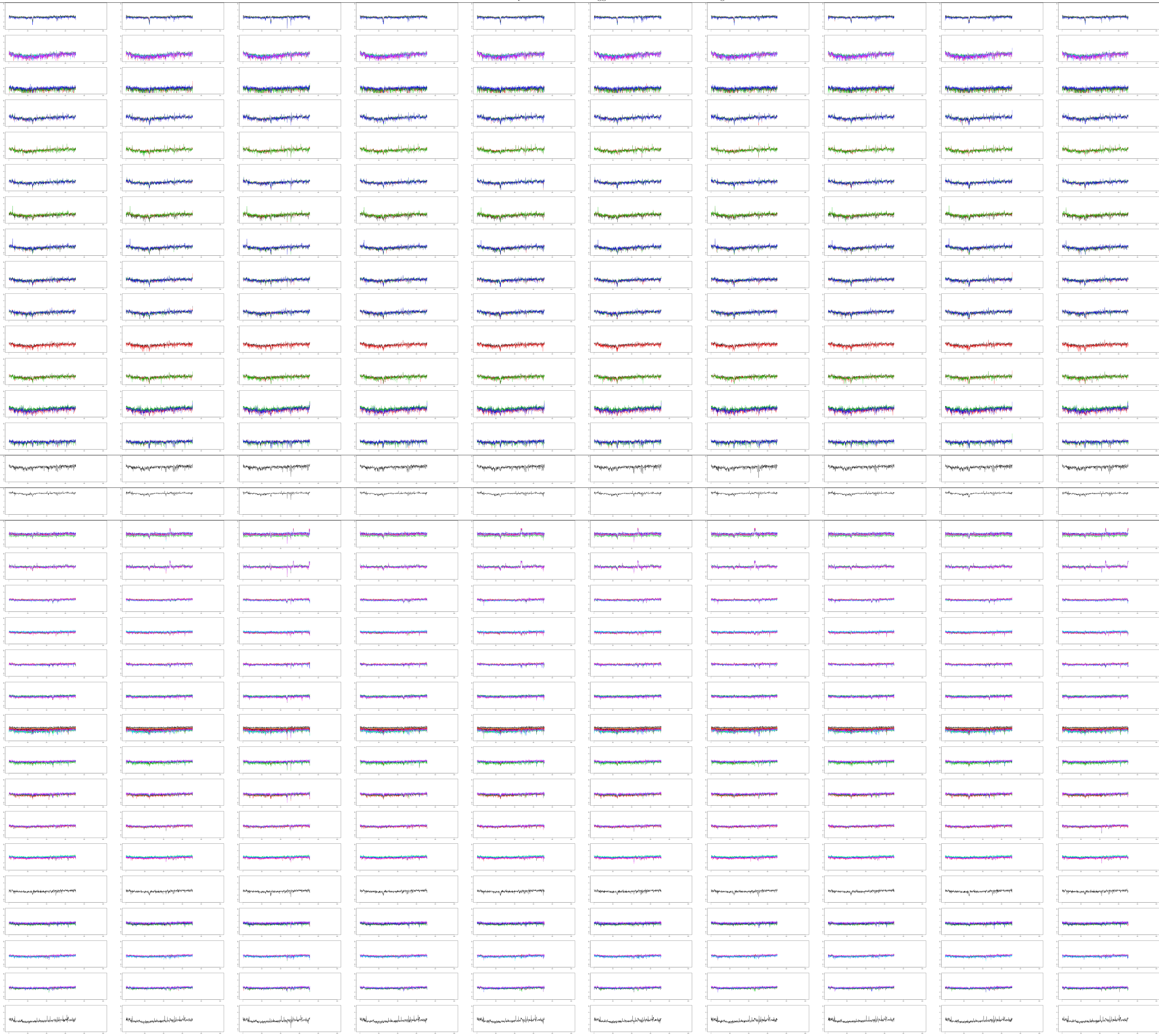

Chromosome 5B

Genome	Species	CS	Julius	ArinaLrFor	CDC_Landmark	CDC_Stanley	Jagger	LongReach_Lancer	Mace	Norin61	SY_Mattis
A	*Triticum urartu*										
S	*Aegilops speltoides*										
D	*Aegilops tauschii*										
C	*Aegilops markgrafii*										
U	*Aegilops umbellulata*										
T	*Aegilops mutica*										
M	*Aegilops comosa*										
N	*Aegilops uniaristata*										
S^l	*Aegilops longissima*										
S^s	*Aegilops searsii*										
S^{sh}	*Aegilops sharonensis*										
S^b	*Aegilops bicornis*										
V	*Dasypyrum villosum*										
Ta	*Taeniatherum caput medusae*										
$EEEE^{st}E^{st}$	*Thinopyrum ponticum*										
DN	*Aegilops ventricosa*										
A^b	*Triticum boeoticum*										
A^b	*Triticum monococcum*										
BA	*Triticum carthlicum*										
BA	*Triticum dicoccoides*										
BA	*Triticum dicoccon*										
BA	*Triticum polonicum*										
BA	*Triticum durum*										
BA	*Triticum turanicum*										
BA	*Triticum turgidum*										
GA^t	*Triticum armeniacum*										
GA^t	*Triticum timopheevii*										
BA^tA^b	*Triticum zhukovskyi*										
BAD	*Triticum spelta*										
BAD	*Triticum sphaerococcum*										
BAD	*Triticum compactum*										
UC	*Aegilops triuncialis*										

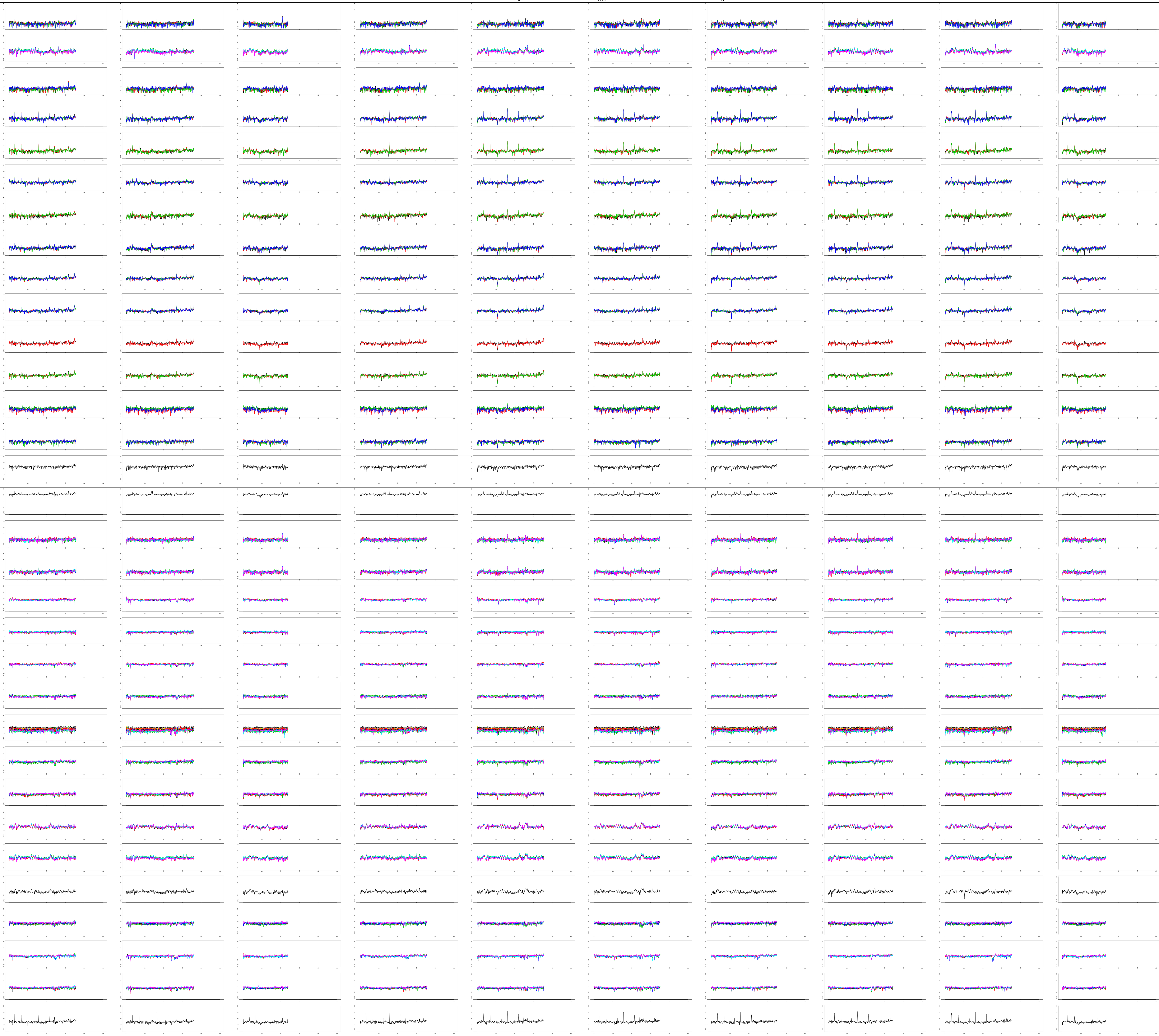

Chromosome 5D

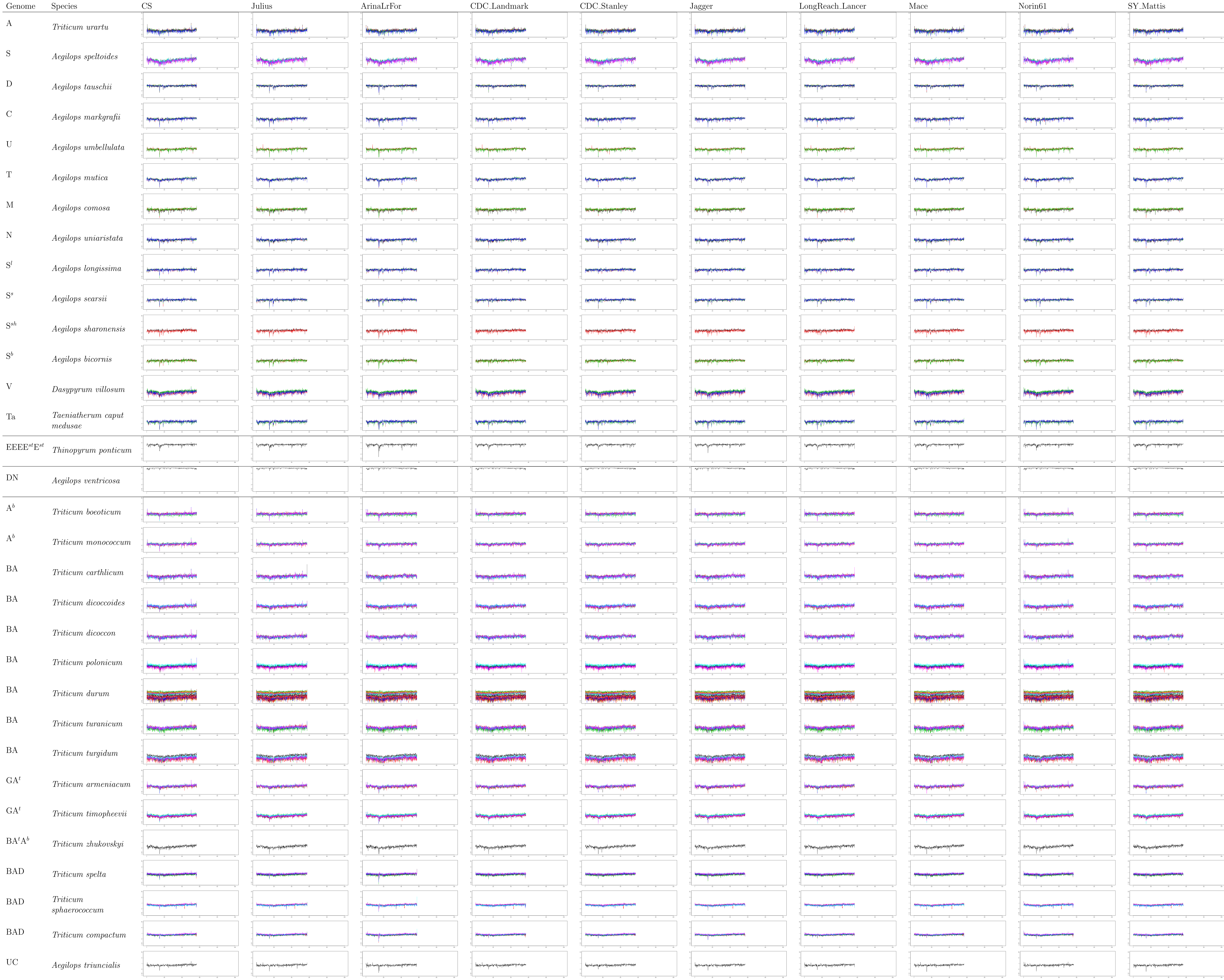

Chromosome 6A

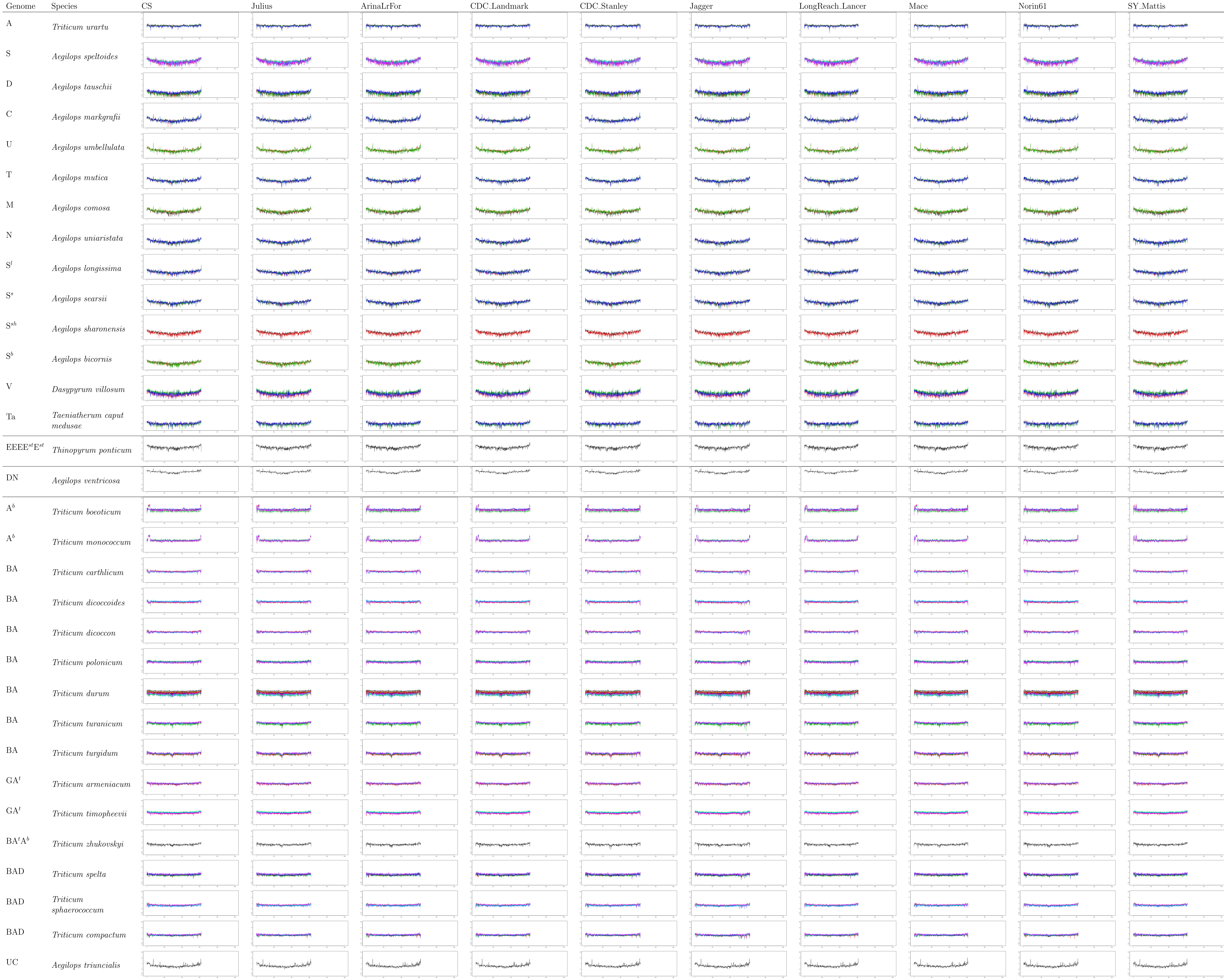

Chromosome 6B

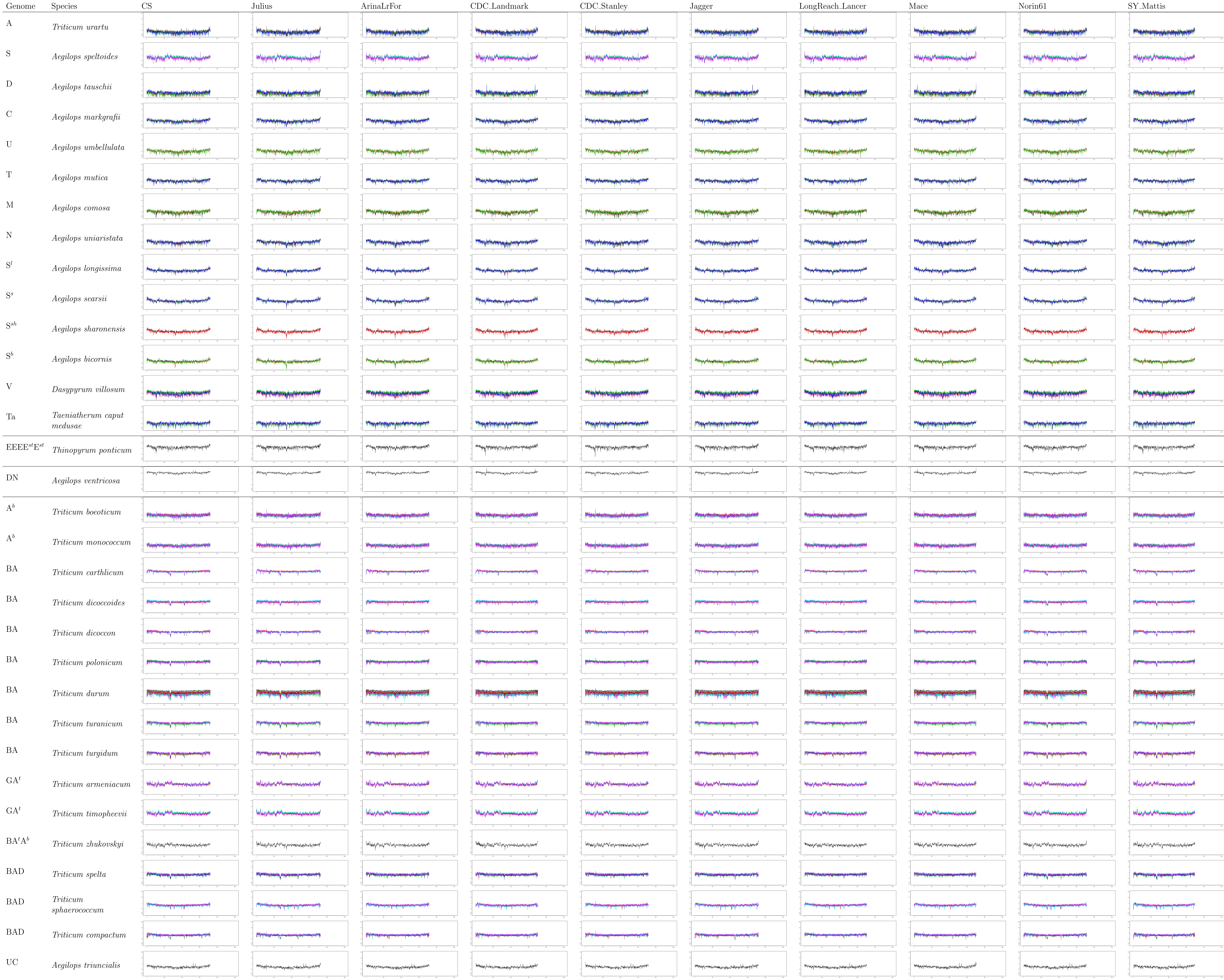

Chromosome 6D

Genome	Species
A	*Triticum urartu*
S	*Aegilops speltoides*
D	*Aegilops tauschii*
C	*Aegilops markgrafii*
U	*Aegilops umbellulata*
T	*Aegilops mutica*
M	*Aegilops comosa*
N	*Aegilops uniaristata*
S^l	*Aegilops longissima*
S^s	*Aegilops searsii*
S^{sh}	*Aegilops sharonensis*
S^b	*Aegilops bicornis*
V	*Dasypyrum villosum*
Ta	*Taeniatherum caput medusae*
$EEEE^{st}E^{st}$	*Thinopyrum ponticum*
DN	*Aegilops ventricosa*
A^b	*Triticum boeoticum*
A^b	*Triticum monococcum*
BA	*Triticum carthlicum*
BA	*Triticum dicoccoides*
BA	*Triticum dicoccon*
BA	*Triticum polonicum*
BA	*Triticum durum*
BA	*Triticum turanicum*
BA	*Triticum turgidum*
GA^t	*Triticum armeniacum*
GA^t	*Triticum timopheevii*
BA^tA^b	*Triticum zhukovskyi*
BAD	*Triticum spelta*
BAD	*Triticum sphaerococcum*
BAD	*Triticum compactum*
UC	*Aegilops triuncialis*

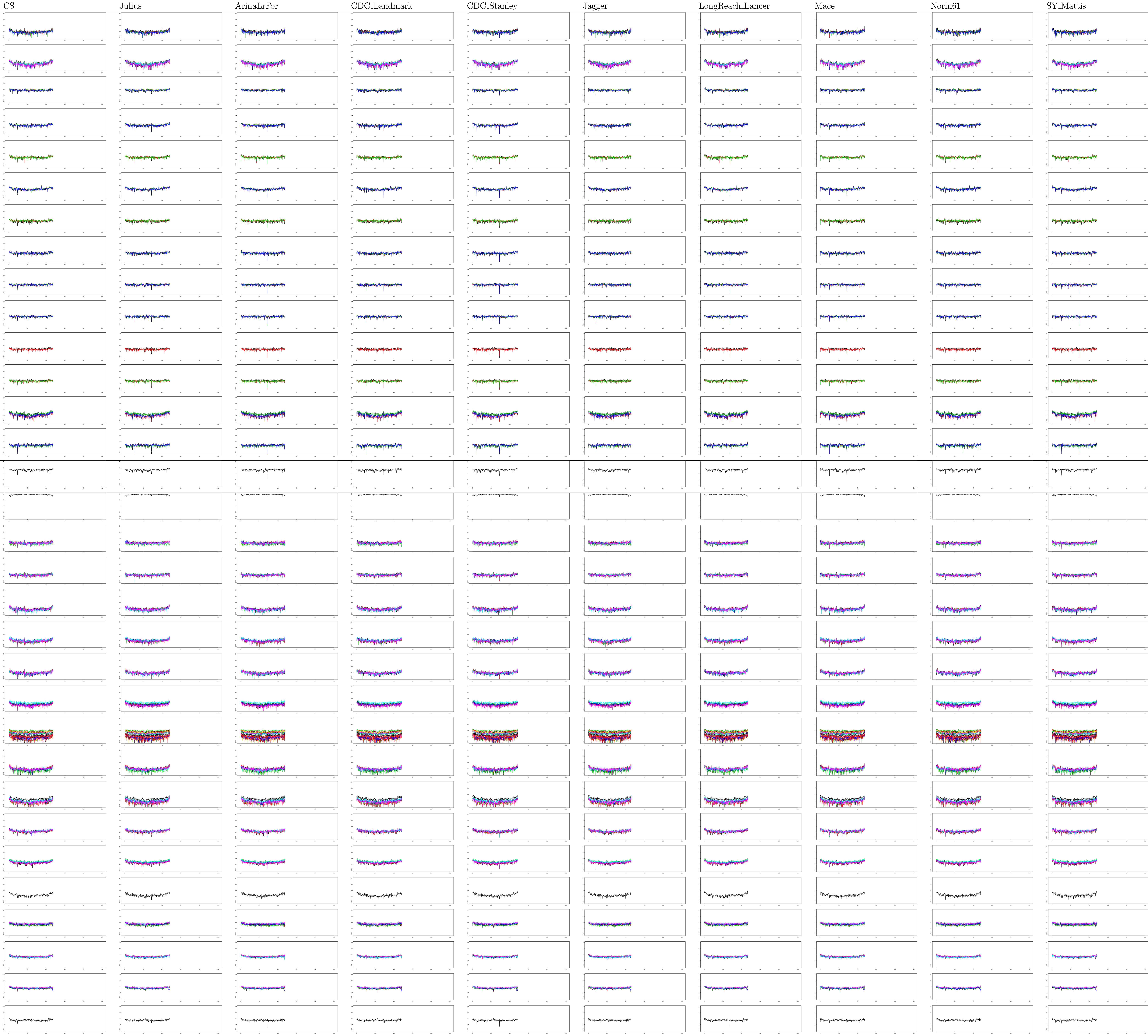

Chromosome 7A

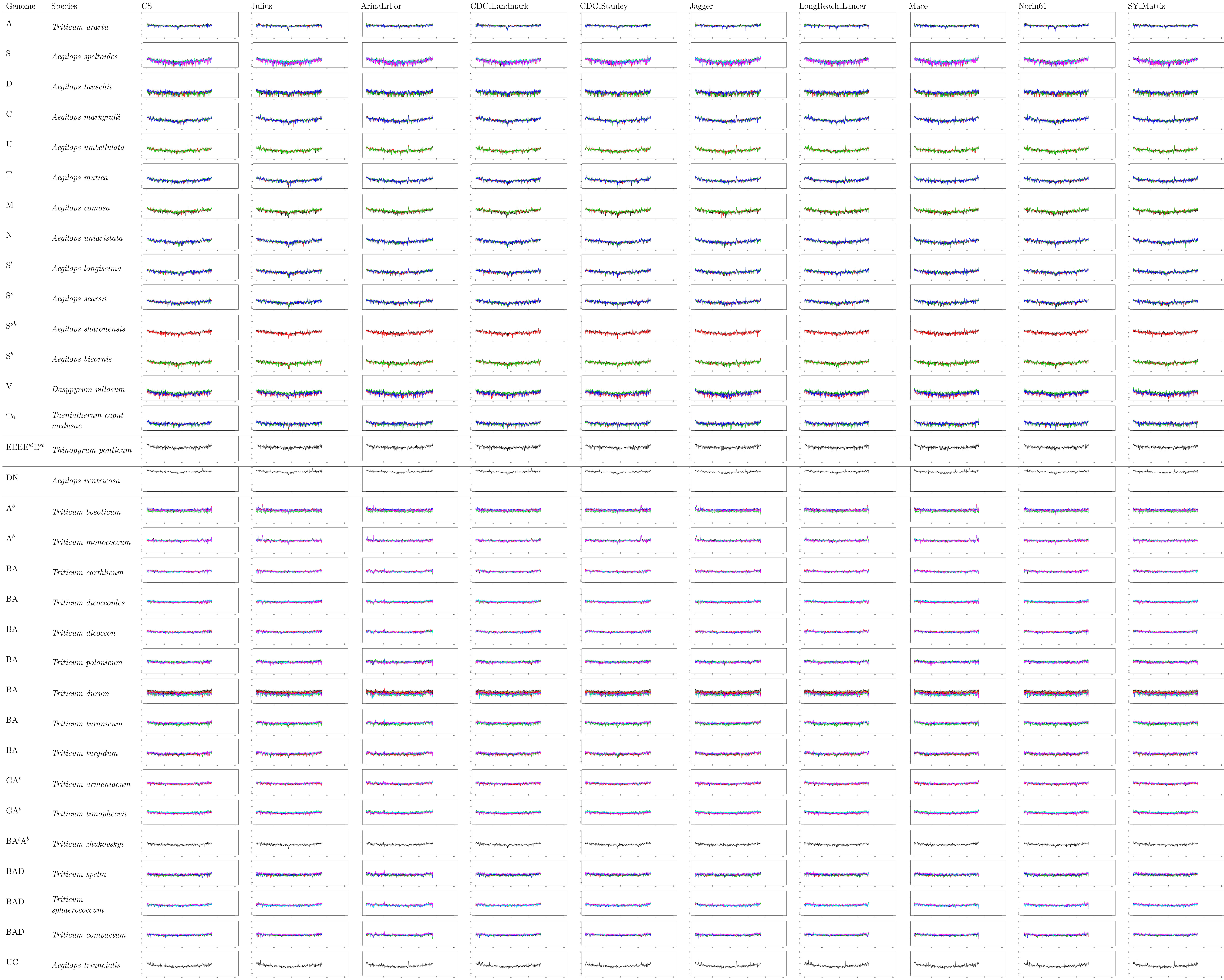

Chromosome 7B

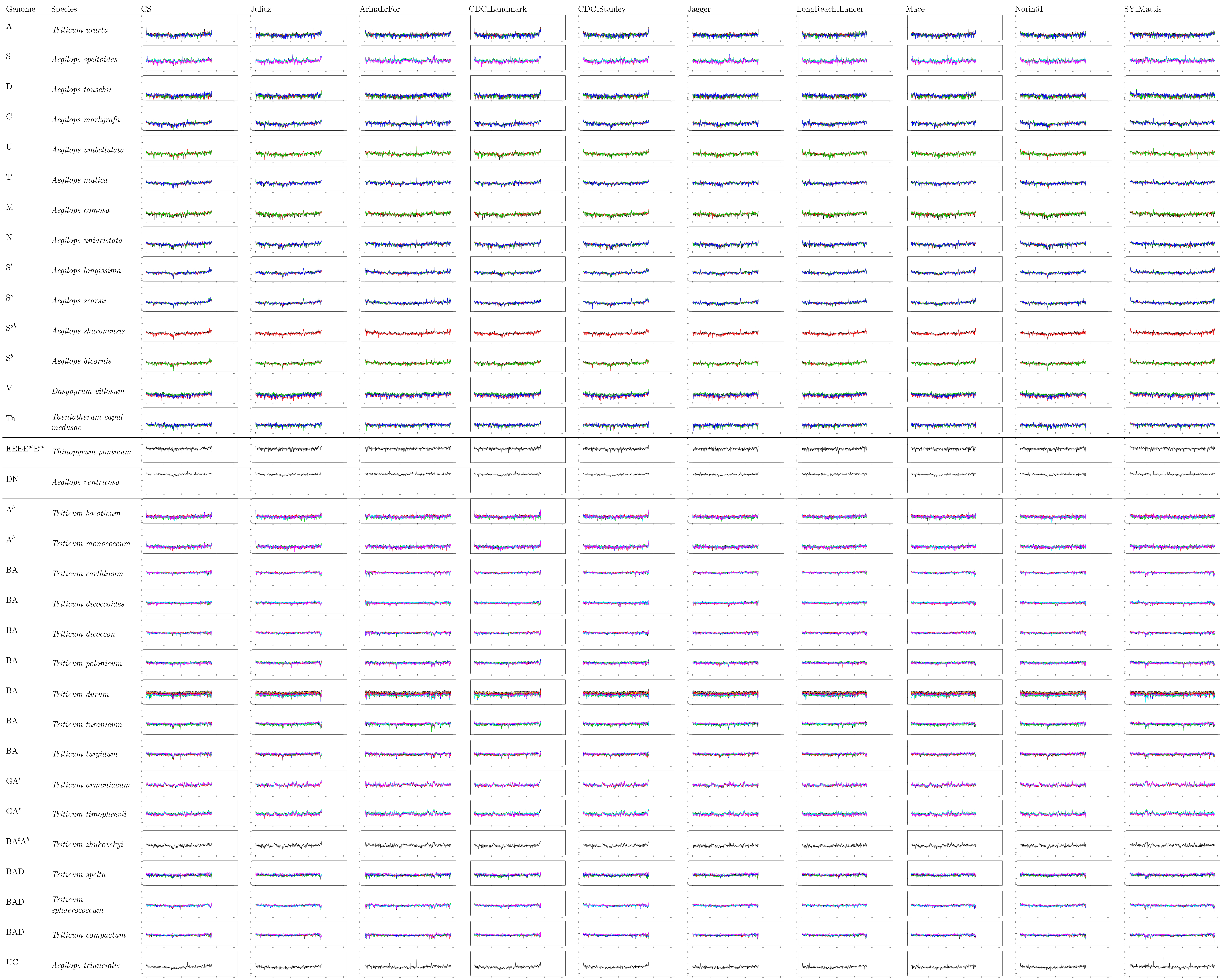

Chromosome 7D

Genome	Species
A	*Triticum urartu*
S	*Aegilops speltoides*
D	*Aegilops tauschii*
C	*Aegilops markgrafii*
U	*Aegilops umbellulata*
T	*Aegilops mutica*
M	*Aegilops comosa*
N	*Aegilops uniaristata*
S^l	*Aegilops longissima*
S^s	*Aegilops searsii*
S^{sh}	*Aegilops sharonensis*
S^b	*Aegilops bicornis*
V	*Dasypyrum villosum*
Ta	*Taeniatherum caput medusae*
$EEEE^{st}E^{st}$	*Thinopyrum ponticum*
DN	*Aegilops ventricosa*
A^b	*Triticum boeoticum*
A^b	*Triticum monococcum*
BA	*Triticum carthlicum*
BA	*Triticum dicoccoides*
BA	*Triticum dicoccon*
BA	*Triticum polonicum*
BA	*Triticum durum*
BA	*Triticum turanicum*
BA	*Triticum turgidum*
GA^t	*Triticum armeniacum*
GA^t	*Triticum timopheevii*
BA^tA^b	*Triticum zhukovskyi*
BAD	*Triticum spelta*
BAD	*Triticum sphaerococcum*
BAD	*Triticum compactum*
UC	*Aegilops triuncialis*

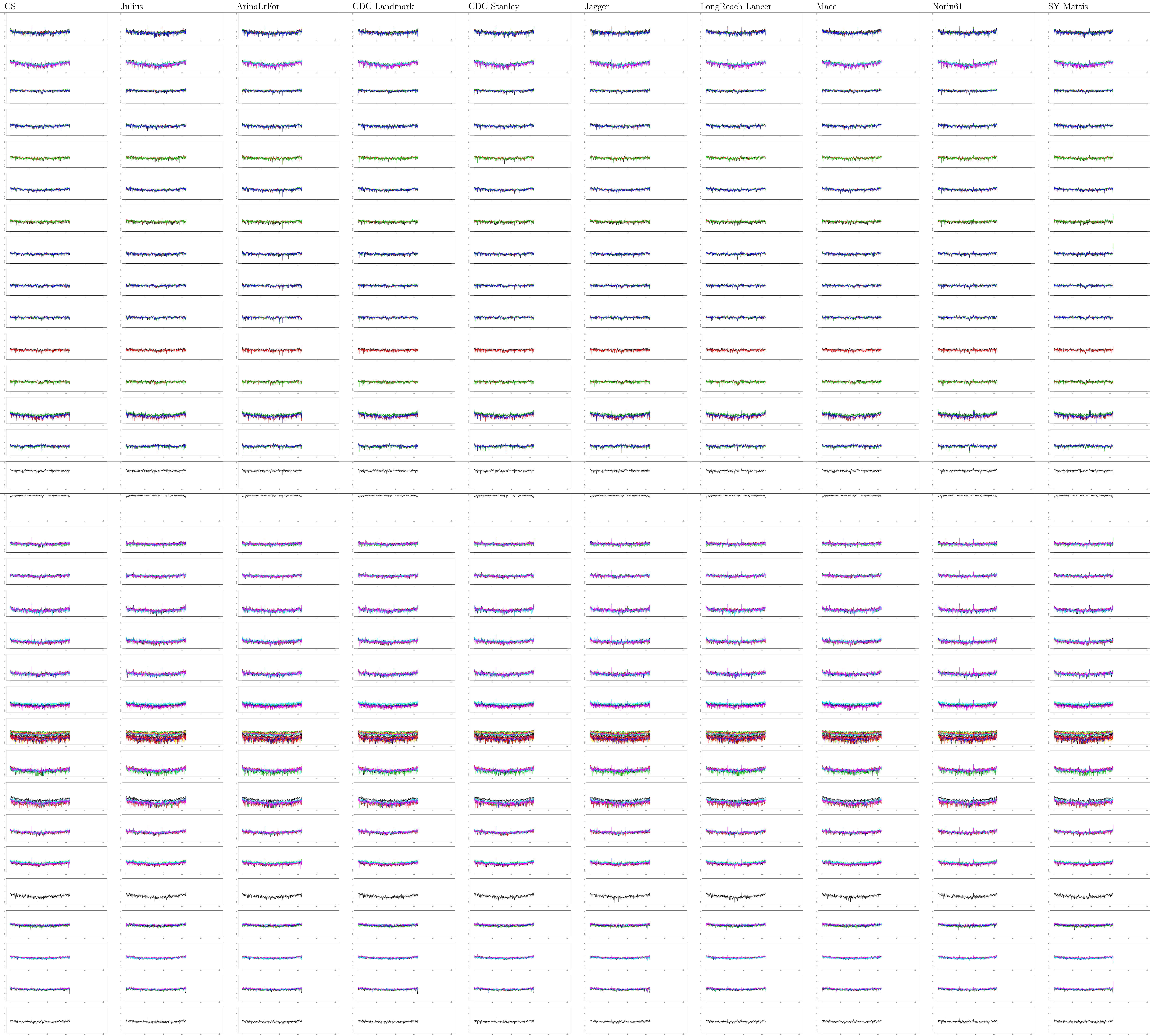

Supplement: Supplementary file 1 — Supplementary Information 1. [file 41598_2022_5865_MOESM1_ESM.pdf]
