# Supplementary material for: Detecting major introgressions in wheat and their putative origins using coverage analysis
Source: Sci Rep. 2022 Feb 3;12:1908. doi: 10.1038/s41598-022-05865-w (PMC8813953; doi:10.1038/s41598-022-05865-w)
Supplement: Supplementary file 2 — Supplementary Information 2. [file 41598_2022_5865_MOESM2_ESM.pdf]

Summary of coverage analysis for eight old wheat cultivars. Columns correspond to chromosomes and rows correspond to accessions. Each plot shows coverage data from four individual plants displayed in four different colours.

Accessions	Chromosome 1A	Heterogenity
TRI 4256<br>Dreadnought<br>1908	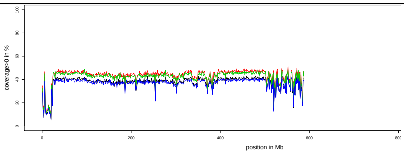	-
TRI 28701<br>Japhet<br>1906	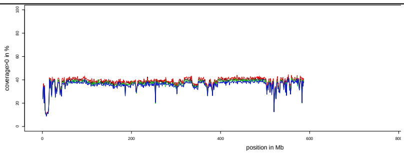	-
TRI 6711<br>Bon Fermier<br>1904	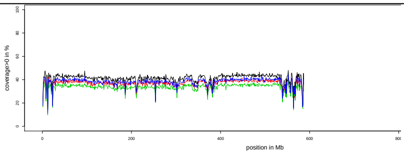	-
TRI 1115<br>Dattel<br>1874	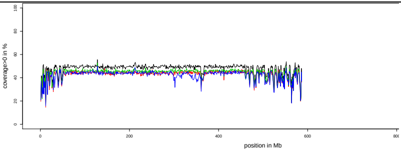	-
TRI 4503<br>Krymka<br>1873	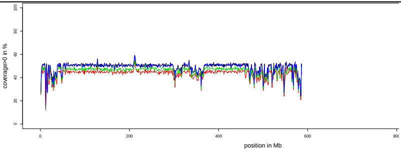	-
TRI 1144<br>Ble Seigle<br>1851	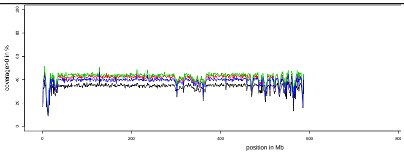	-
TRI 208<br>Gros Bleu<br>1837	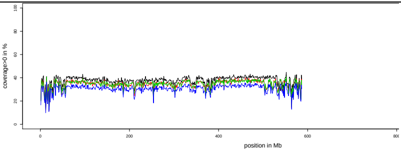	1AL
TRI 782<br>Noe<br>1826	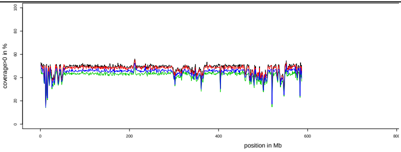	-

Accessions	Chromosome 1B	Heterogenity
TRI 4256<br>Dreadnought<br>1908	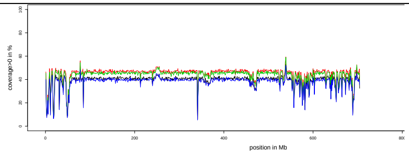	-
TRI 28701<br>Japhet<br>1906	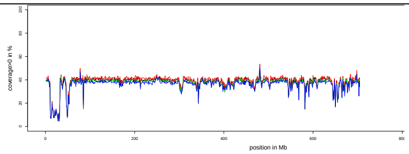	-
TRI 6711<br>Bon Fermier<br>1904	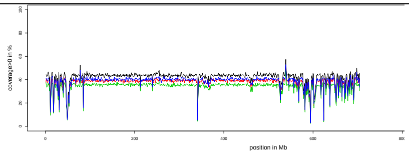	-
TRI 1115<br>Dattel<br>1874	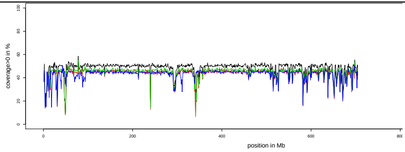	1B
TRI 4503<br>Krymka<br>1873	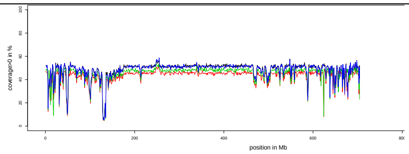	-
TRI 1144<br>Ble Seigle<br>1851	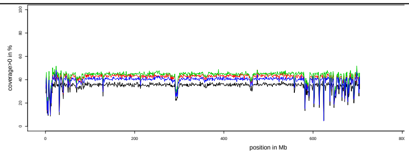	-
TRI 208<br>Gros Bleu<br>1837	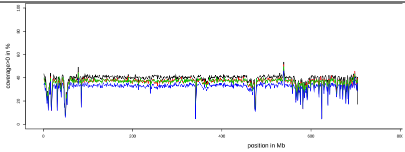	-
TRI 782<br>Noe<br>1826	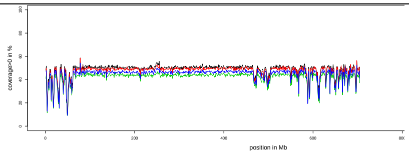	-

Accessions	Chromosome 1D	Heterogenity
TRI 4256<br>Dreadnought<br>1908	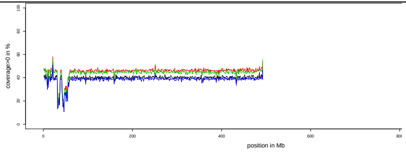	-
TRI 28701<br>Japhet<br>1906	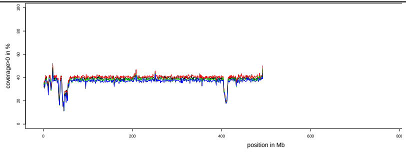	-
TRI 6711<br>Bon Fermier<br>1904	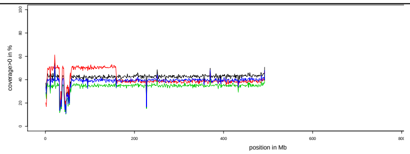	1DS
TRI 1115<br>Dattel<br>1874	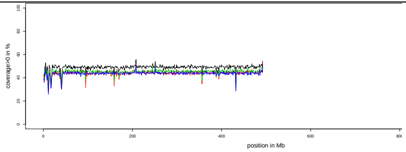	-
TRI 4503<br>Krymka<br>1873	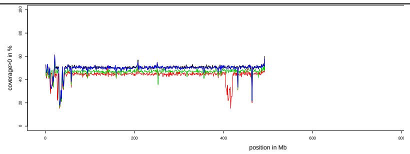	1DS, 1DL
TRI 1144<br>Ble Seigle<br>1851	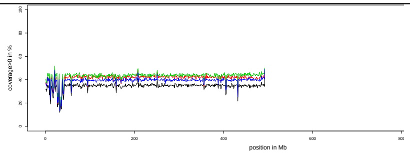	-
TRI 208<br>Gros Bleu<br>1837	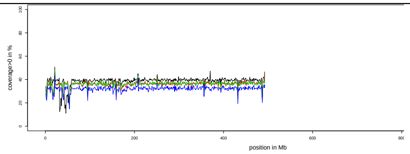	1DS
TRI 782<br>Noe<br>1826	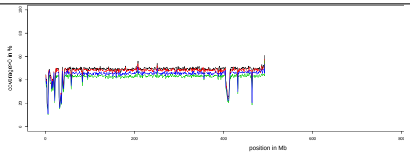	-

Accessions	Chromosome 2A	Heterogenity
TRI 4256<br>Dreadnought<br>1908	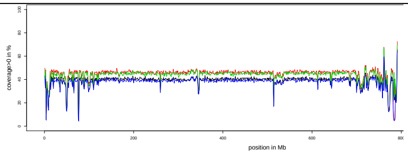	-
TRI 28701<br>Japhet<br>1906	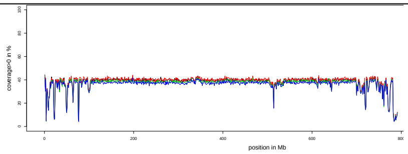	-
TRI 6711<br>Bon Fermier<br>1904	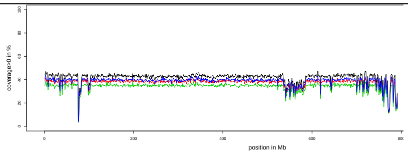	-
TRI 1115<br>Dattel<br>1874	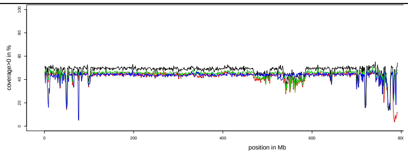	2AL
TRI 4503<br>Krymka<br>1873	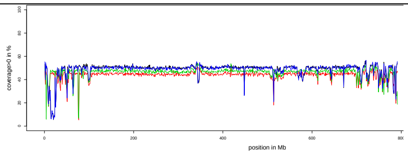	2AS, 2AL
TRI 1144<br>Ble Seigle<br>1851	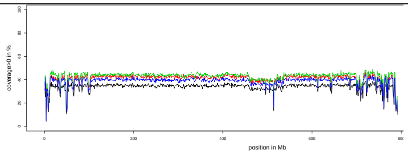	-
TRI 208<br>Gros Bleu<br>1837	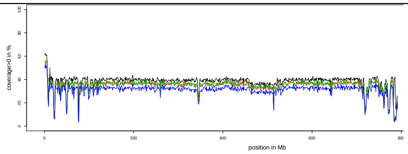	-
TRI 782<br>Noe<br>1826	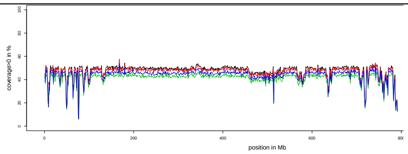	-

Accessions	Chromosome 2B	Heterogenity
TRI 4256 Dreadnought 1908	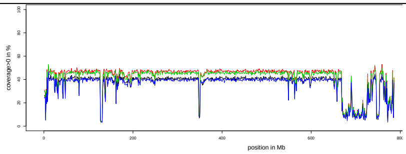	-
TRI 28701 Japhet 1906	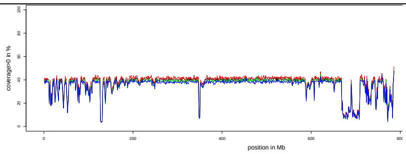	-
TRI 6711 Bon Fermier 1904	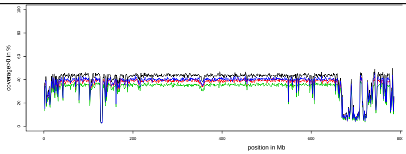	-
TRI 1115 Dattel 1874	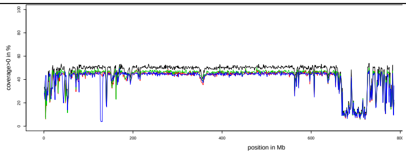	2BS
TRI 4503 Krymka 1873	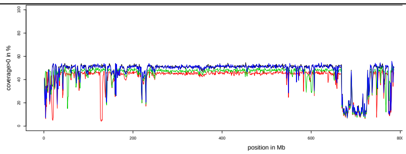	2BS, 2BL
TRI 1144 Ble Seigle 1851	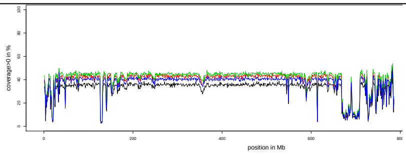	-
TRI 208 Gros Bleu 1837	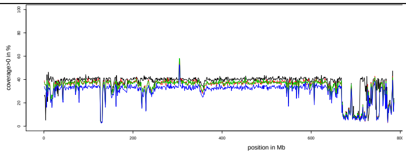	2BS, 2BL
TRI 782 Noe 1826	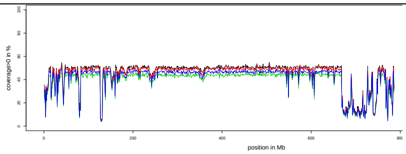	-

Accessions	Chromosome 2D	Heterogenity
TRI 4256 Dreadnought 1908	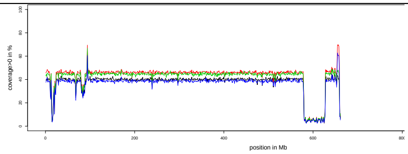	-
TRI 28701 Japhet 1906	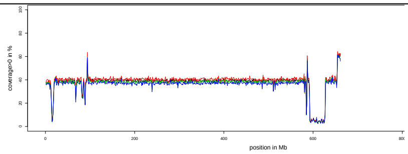	-
TRI 6711 Bon Fermier 1904	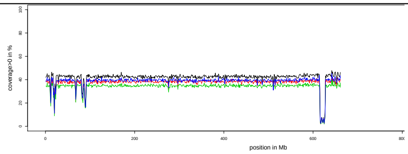	-
TRI 1115 Dattel 1874	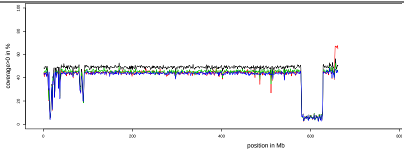	2DL
TRI 4503 Krymka 1873	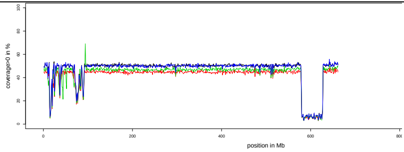	2DS
TRI 1144 Ble Seigle 1851	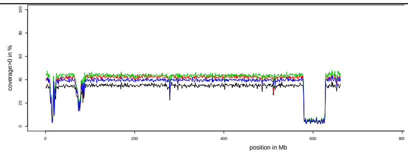	-
TRI 208 Gros Bleu 1837	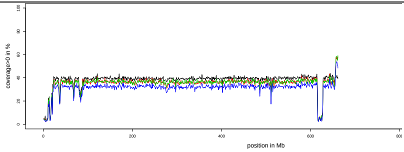	2DL
TRI 782 Noe 1826	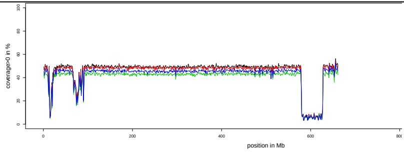	-

Accessions	Chromosome 3A	Heterogenity
TRI 4256<br>Dreadnought<br>1908	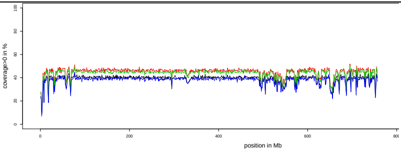	-
TRI 28701<br>Japhet<br>1906	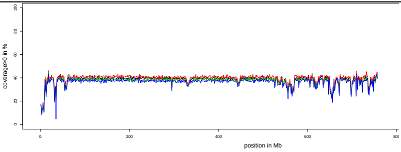	-
TRI 6711<br>Bon Fermier<br>1904	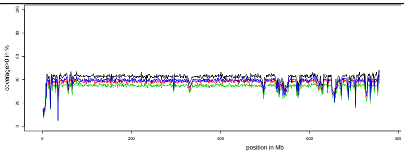	-
TRI 1115<br>Dattel<br>1874	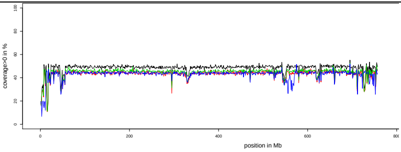	3AS,3AL
TRI 4503<br>Krymka<br>1873	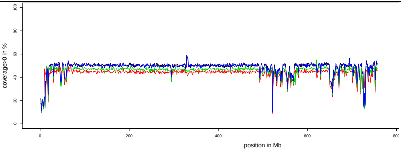	3A
TRI 1144<br>Ble Seigle<br>1851	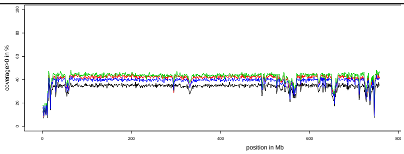	-
TRI 208<br>Gros Bleu<br>1837	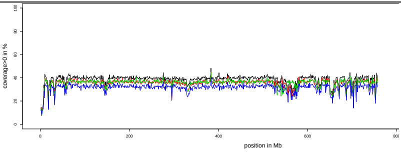	3AL
TRI 782<br>Noe<br>1826	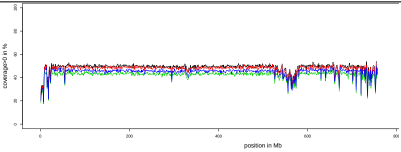	-

Accessions	Chromosome 3B	Heterogenity
TRI 4256 Dreadnought 1908	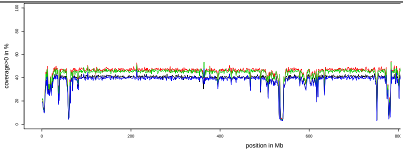	-
TRI 28701 Japhet 1906	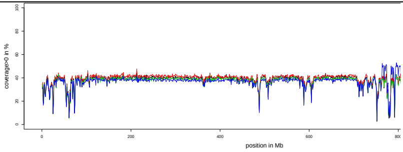	3BL
TRI 6711 Bon Fermier 1904	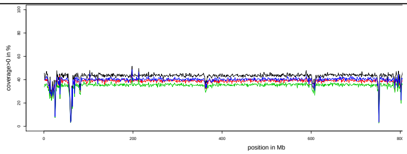	-
TRI 1115 Dattel 1874	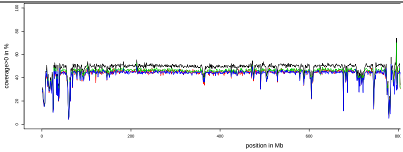	3BL
TRI 4503 Krymka 1873	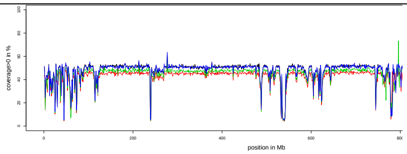	3BL
TRI 1144 Ble Seigle 1851	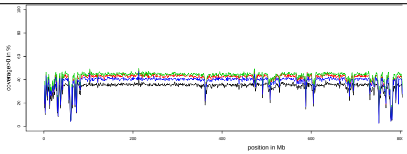	-
TRI 208 Gros Bleu 1837	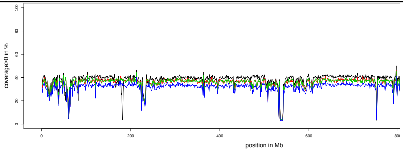	-
TRI 782 Noe 1826	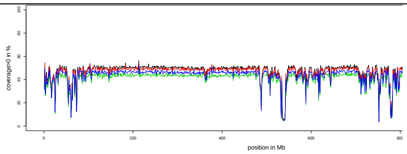	-

Accessions	Chromosome 3D	Heterogenity
TRI 4256 Dreadnought 1908	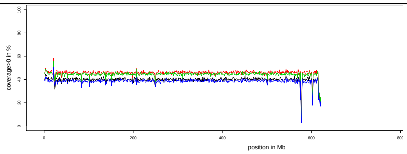	-
TRI 28701 Japhet 1906	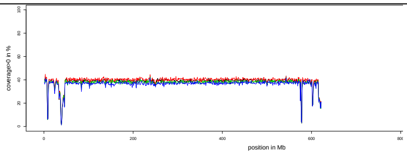	-
TRI 6711 Bon Fermier 1904	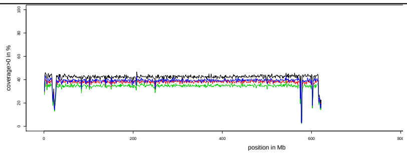	-
TRI 1115 Dattel 1874	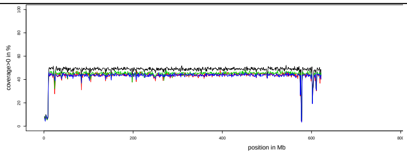	-
TRI 4503 Krymka 1873	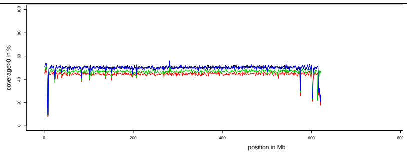	3DL
TRI 1144 Ble Seigle 1851	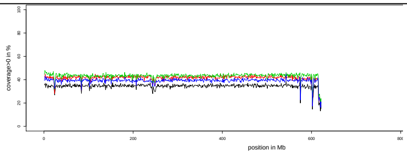	-
TRI 208 Gros Bleu 1837	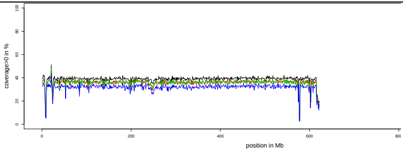	-
TRI 782 Noe 1826	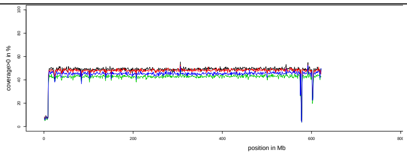	-

Accessions	Chromosome 4A	Heterogenity
TRI 4256 Dreadnought 1908	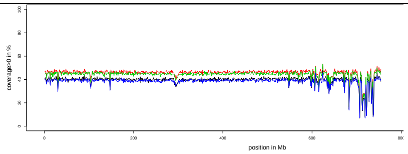	-
TRI 28701 Japhet 1906	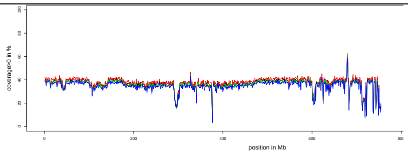	-
TRI 6711 Bon Fermier 1904	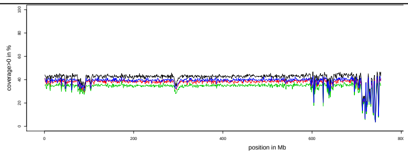	-
TRI 1115 Dattel 1874	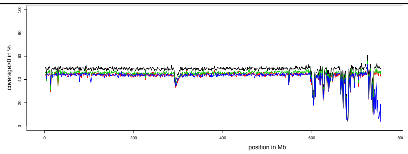	4AL
TRI 4503 Krymka 1873	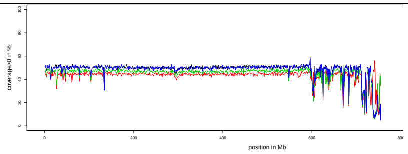	4AL
TRI 1144 Ble Seigle 1851	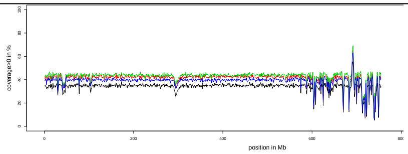	-
TRI 208 Gros Bleu 1837	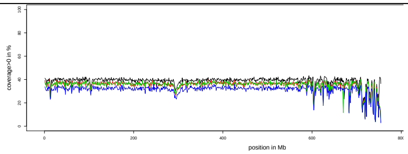	4AL
TRI 782 Noe 1826	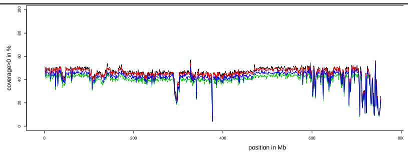	-

Accessions	Chromosome 4B	Heterogenity
TRI 4256<br>Dreadnought<br>1908	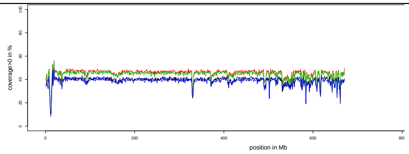	-
TRI 28701<br>Japhet<br>1906	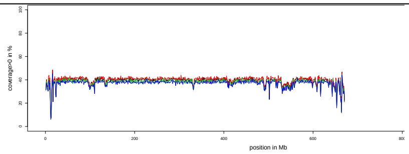	-
TRI 6711<br>Bon Fermier<br>1904	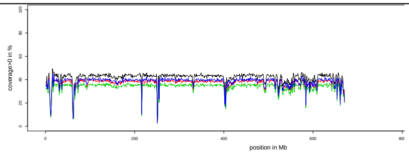	-
TRI 1115<br>Dattel<br>1874	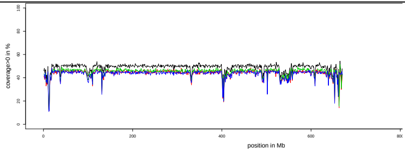	-
TRI 4503<br>Krymka<br>1873	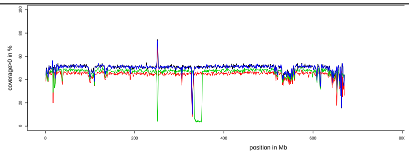	4B
TRI 1144<br>Ble Seigle<br>1851	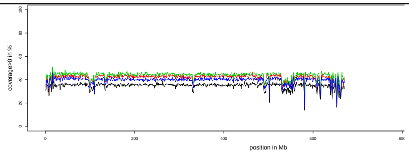	-
TRI 208<br>Gros Bleu<br>1837	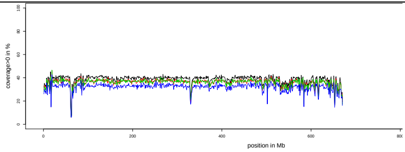	-
TRI 782<br>Noe<br>1826	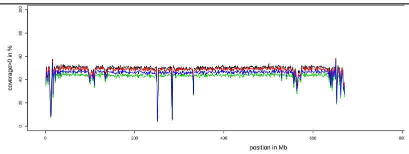	-

Accessions	Chromosome 4D	Heterogenity
TRI 4256<br>Dreadnought<br>1908	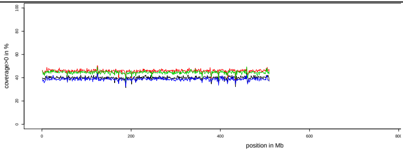	-
TRI 28701<br>Japhet<br>1906	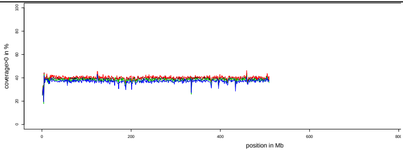	-
TRI 6711<br>Bon Fermier<br>1904	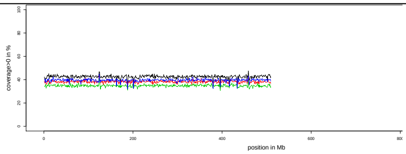	-
TRI 1115<br>Dattel<br>1874	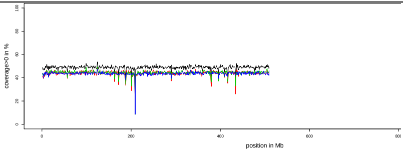	-
TRI 4503<br>Krymka<br>1873	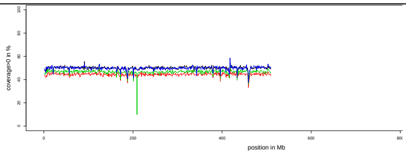	4D
TRI 1144<br>Ble Seigle<br>1851	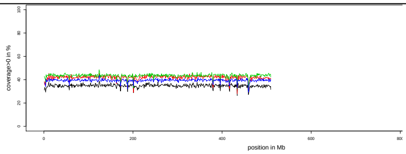	-
TRI 208<br>Gros Bleu<br>1837	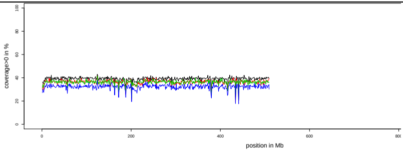	-
TRI 782<br>Noe<br>1826	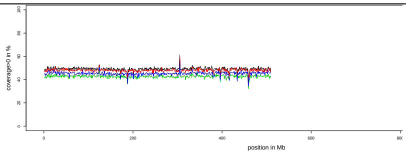	-

Accessions	Chromosome 5A	Heterogenity
TRI 4256<br>Dreadnought<br>1908	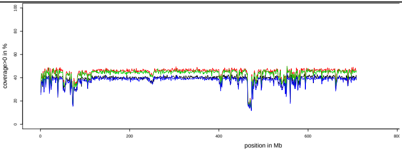	-
TRI 28701<br>Japhet<br>1906	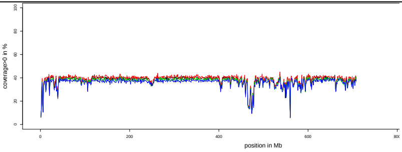	-
TRI 6711<br>Bon Fermier<br>1904	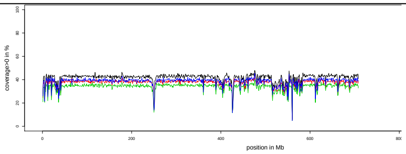	-
TRI 1115<br>Dattel<br>1874	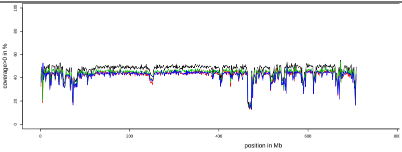	5AS
TRI 4503<br>Krymka<br>1873	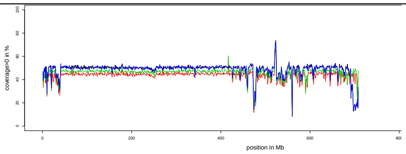	5AL
TRI 1144<br>Ble Seigle<br>1851	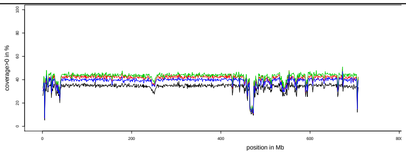	-
TRI 208<br>Gros Bleu<br>1837	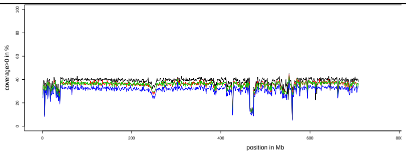	-
TRI 782<br>Noe<br>1826	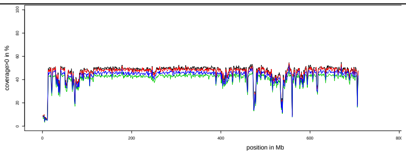	-

Accessions	Chromosome 5B	Heterogenity
TRI 4256<br>Dreadnought<br>1908	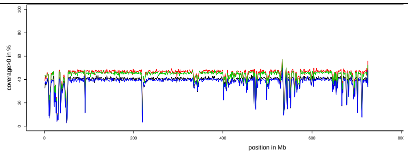	-
TRI 28701<br>Japhet<br>1906	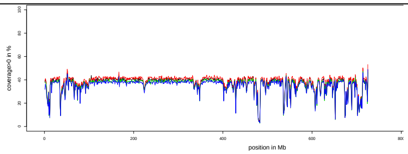	-
TRI 6711<br>Bon Fermier<br>1904	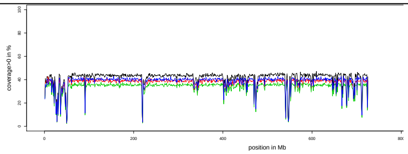	-
TRI 1115<br>Dattel<br>1874	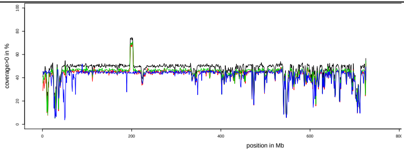	5BS
TRI 4503<br>Krymka<br>1873	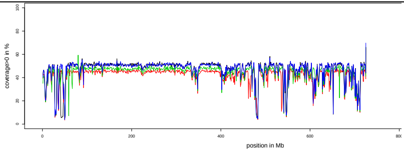	5BS, 5BL
TRI 1144<br>Ble Seigle<br>1851	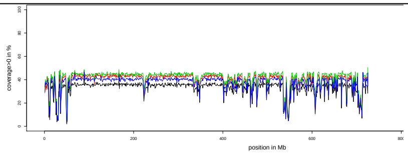	-
TRI 208<br>Gros Bleu<br>1837	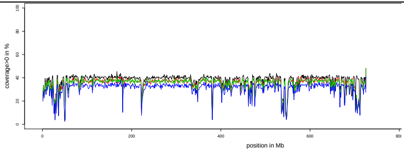	5BL
TRI 782<br>Noe<br>1826	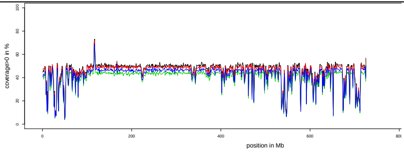	-

Accessions	Chromosome 5D	Heterogenity
TRI 4256 Dreadnought 1908	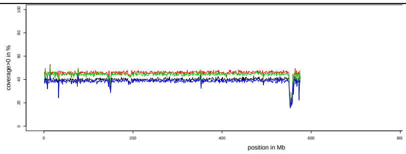	-
TRI 28701 Japhet 1906	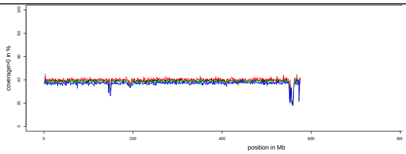	-
TRI 6711 Bon Fermier 1904	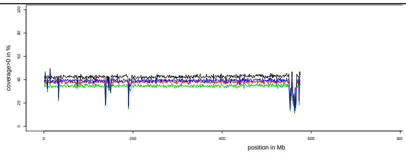	-
TRI 1115 Dattel 1874	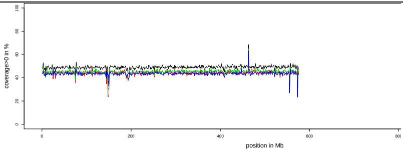	-
TRI 4503 Krymka 1873	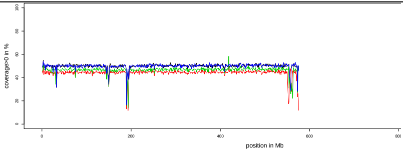	5DL
TRI 1144 Ble Seigle 1851	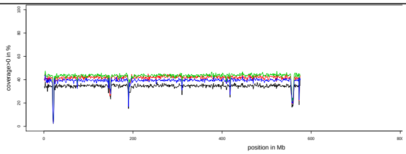	-
TRI 208 Gros Bleu 1837	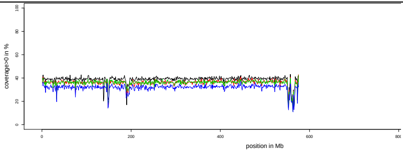	-
TRI 782 Noe 1826	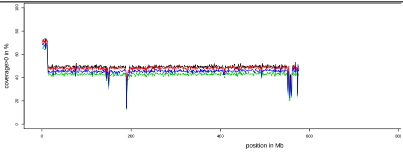	-

Accessions	Chromosome 6A	Heterogenity
TRI 4256<br>Dreadnought<br>1908	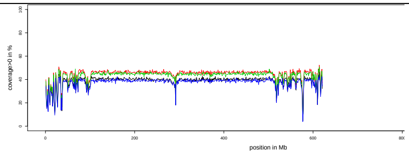	-
TRI 28701<br>Japhet<br>1906	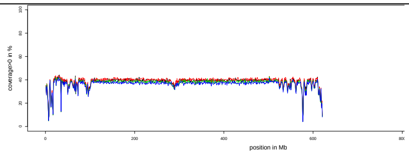	-
TRI 6711<br>Bon Fermier<br>1904	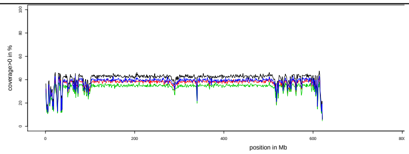	-
TRI 1115<br>Dattel<br>1874	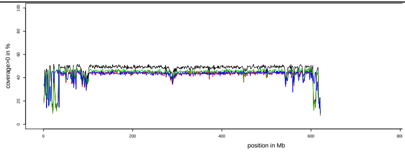	6AS, 6AL
TRI 4503<br>Krymka<br>1873	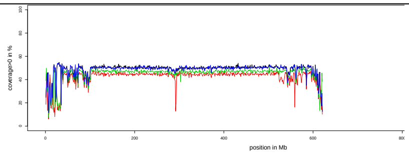	6AS, 6AL
TRI 1144<br>Ble Seigle<br>1851	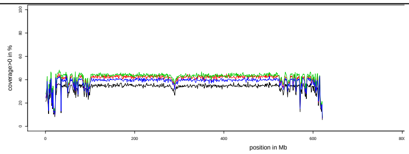	-
TRI 208<br>Gros Bleu<br>1837	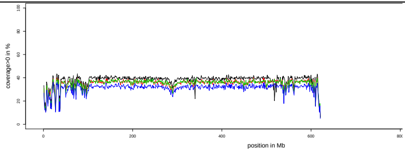	-
TRI 782<br>Noe<br>1826	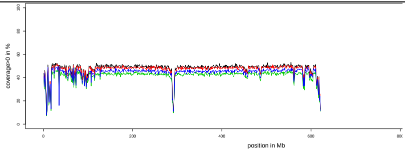	-

Accessions	Chromosome 6B	Heterogenity
TRI 4256<br>Dreadnought<br>1908	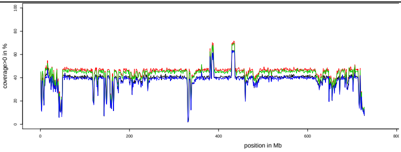	-
TRI 28701<br>Japhet<br>1906	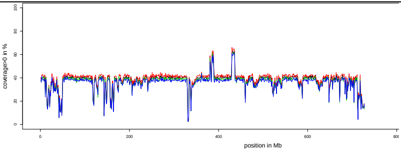	-
TRI 6711<br>Bon Fermier<br>1904	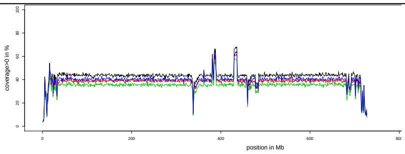	-
TRI 1115<br>Dattel<br>1874	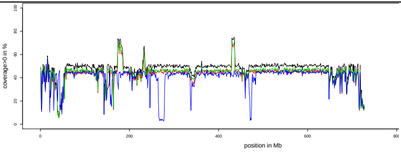	6BS, 6BL
TRI 4503<br>Krymka<br>1873	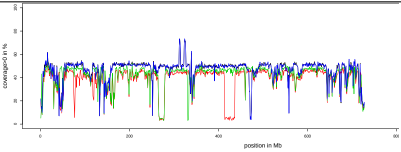	6BS, 6BL
TRI 1144<br>Ble Seigle<br>1851	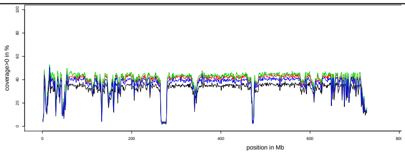	-
TRI 208<br>Gros Bleu<br>1837	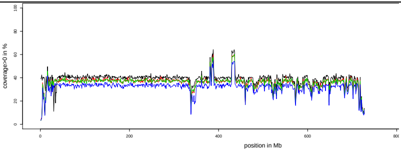	6BS
TRI 782<br>Noe<br>1826	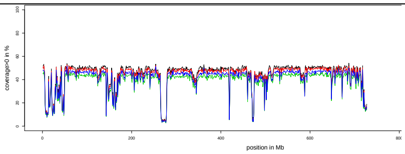	-

Accessions	Chromosome 6D	Heterogenity
TRI 4256<br>Dreadnought<br>1908	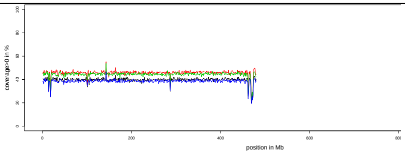	-
TRI 28701<br>Japhet<br>1906	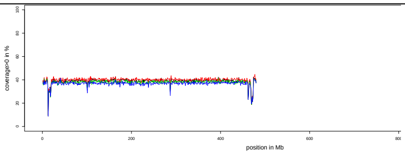	-
TRI 6711<br>Bon Fermier<br>1904	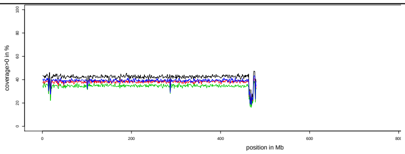	-
TRI 1115<br>Dattel<br>1874	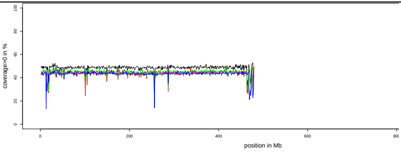	6DS, 6DL
TRI 4503<br>Krymka<br>1873	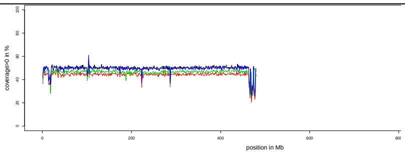	6DS, 6DL
TRI 1144<br>Ble Seigle<br>1851	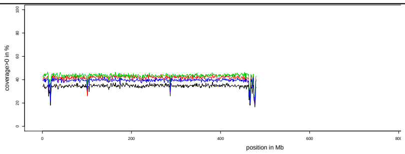	-
TRI 208<br>Gros Bleu<br>1837	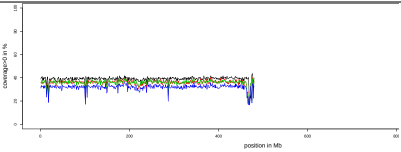	6DL
TRI 782<br>Noe<br>1826	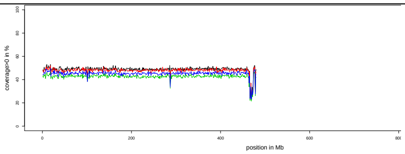	-

Accessions	Chromosome 7A	Heterogenity
TRI 4256<br>Dreadnought<br>1908	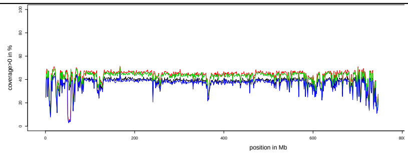	-
TRI 28701<br>Japhet<br>1906	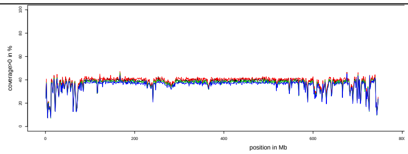	-
TRI 6711<br>Bon Fermier<br>1904	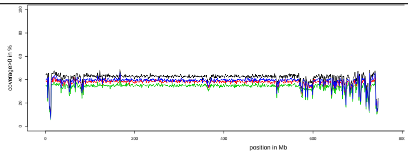	-
TRI 1115<br>Dattel<br>1874	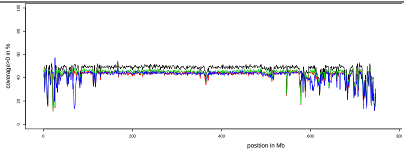	7AS, 7AL
TRI 4503<br>Krymka<br>1873	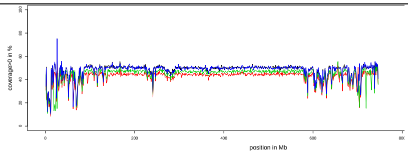	7AS, 7AL
TRI 1144<br>Ble Seigle<br>1851	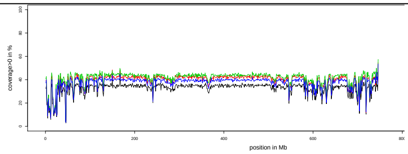	-
TRI 208<br>Gros Bleu<br>1837	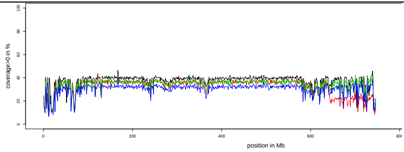	7AL
TRI 782<br>Noe<br>1826	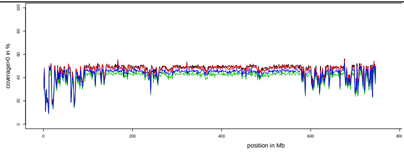	-

<table>
<tr><th>Accessions</th><th>Chromosome 7B</th><th>Heterogenity</th></tr>
<tr><td>TRI 4256
Dreadnought
1908</td><td>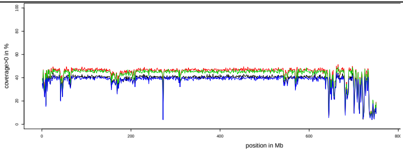
</td><td>-</td></tr>
<tr><td>TRI 28701
Japhet
1906</td><td>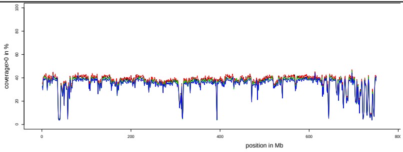
</td><td>-</td></tr>
<tr><td>TRI 6711
Bon Fermier
1904</td><td>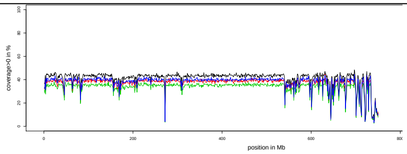
</td><td>-</td></tr>
<tr><td>TRI 1115
Dattel
1874</td><td>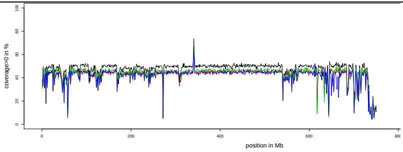
</td><td>7BL</td></tr>
<tr><td>TRI 4503
Krymka
1873</td><td>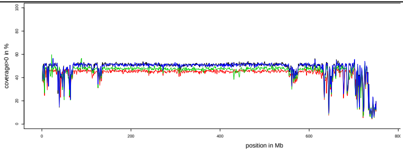
</td><td>7BS, 7BL</td></tr>
<tr><td>TRI 1144
Ble Seigle
1851</td><td>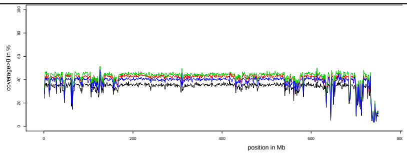
</td><td>-</td></tr>
<tr><td>TRI 208
Gros Bleu
1837</td><td>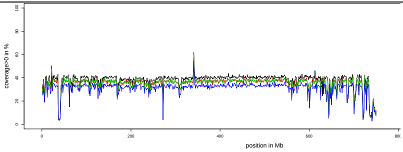
</td><td>7BL</td></tr>
<tr><td>TRI 782
Noe
1826</td><td>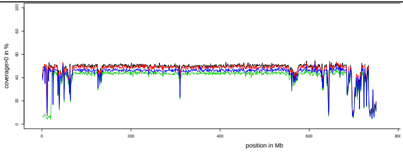
</td><td>7BS</td></tr>
</table>

Accessions	Chromosome 7D	Heterogenity
TRI 4256<br>Dreadnought<br>1908	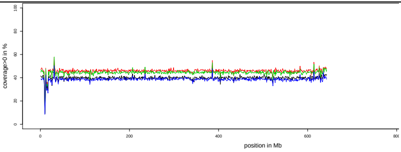	-
TRI 28701<br>Japhet<br>1906	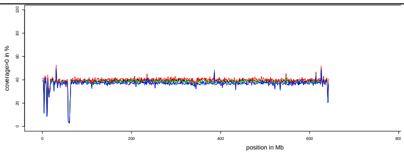	-
TRI 6711<br>Bon Fermier<br>1904	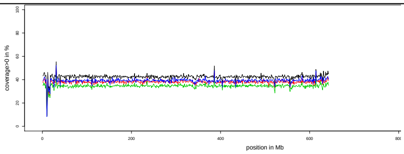	-
TRI 1115<br>Dattel<br>1874	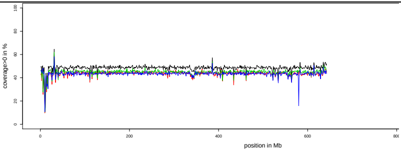	7DL
TRI 4503<br>Krymka<br>1873	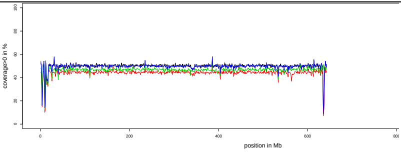	7DL
TRI 1144<br>Ble Seigle<br>1851	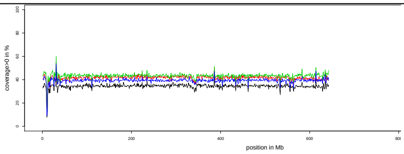	-
TRI 208<br>Gros Bleu<br>1837	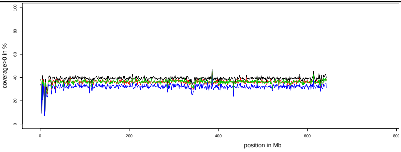	-
TRI 782<br>Noe<br>1826	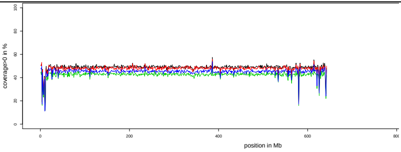	-
