# Supplementary material for: Detecting major introgressions in wheat and their putative origins using coverage analysis
Source: Sci Rep. 2022 Feb 3;12:1908. doi: 10.1038/s41598-022-05865-w (PMC8813953; doi:10.1038/s41598-022-05865-w)
Supplement: Supplementary file 3 — Supplementary Figures. [file 41598_2022_5865_MOESM3_ESM.pdf]

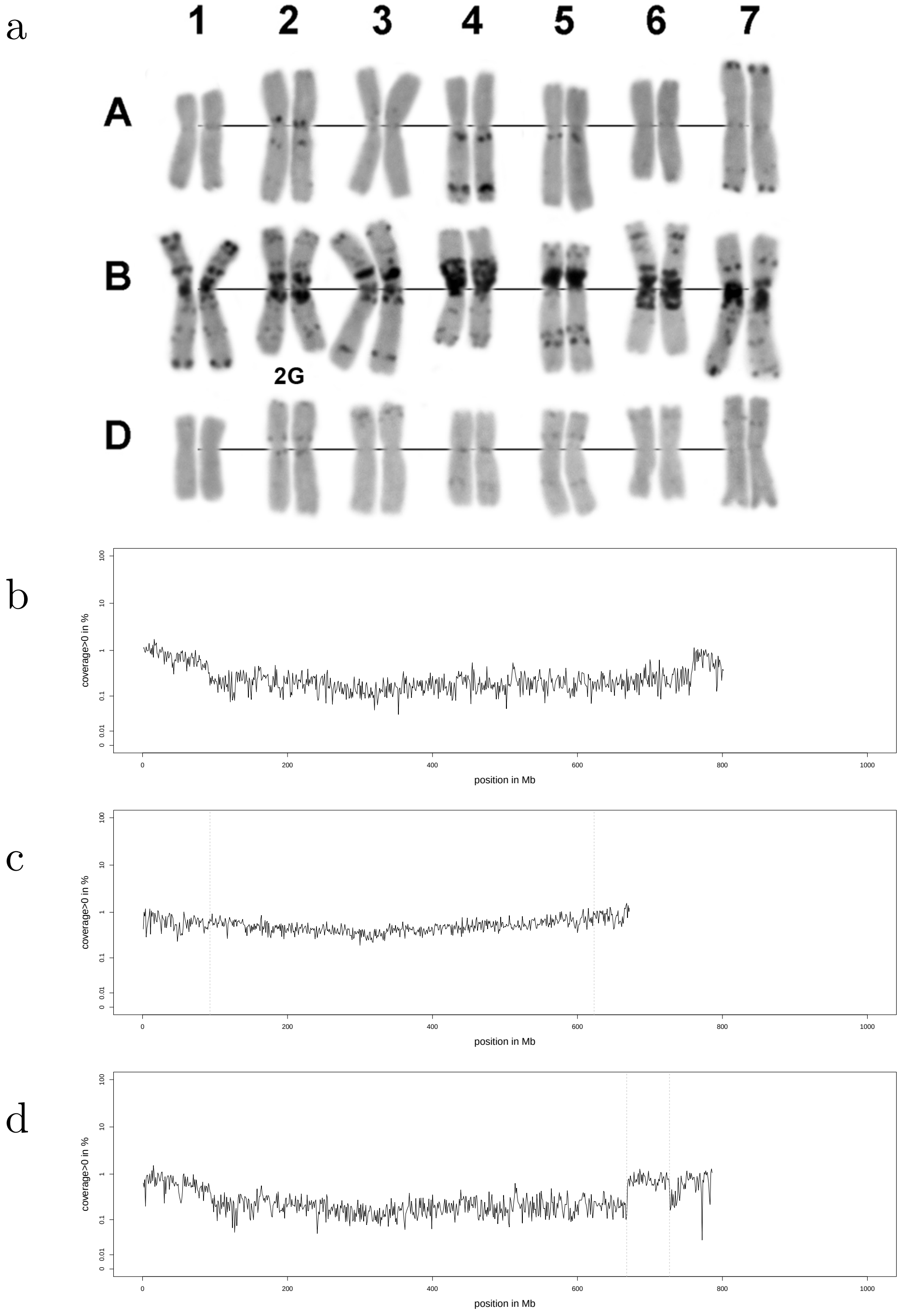

Fig. S1. Karyotype and coverage profiles of cv. Wisconsin-245. a) Karyotype of cv. Wisconsin-245. **b-d)** coverage profiles of chromosome 2B for Wisconsin-245 using cv. Chinese Spring, cv. LongReach Lancer, and cv. Julius as reference genomes. Dashed lines in panel c and d mark borders of the described introgressions 4 and 5. Normal coverage in these regions indicates that the sequence in cv. Wisconsin-245 is similar to that in cv. LongReach Lancer and cv. Julius. Hence, the introgression in cv. Wisconsin-245 might be the origin of the introgressions in cv. LongReach Lancer and cv. Julius. Furthermore, the coverage profile using cv. LongReach Lancer as reference genome shows that the size of the introgressions in cv. Wisconsin-245 and cv. LongReach Lancer is identical.

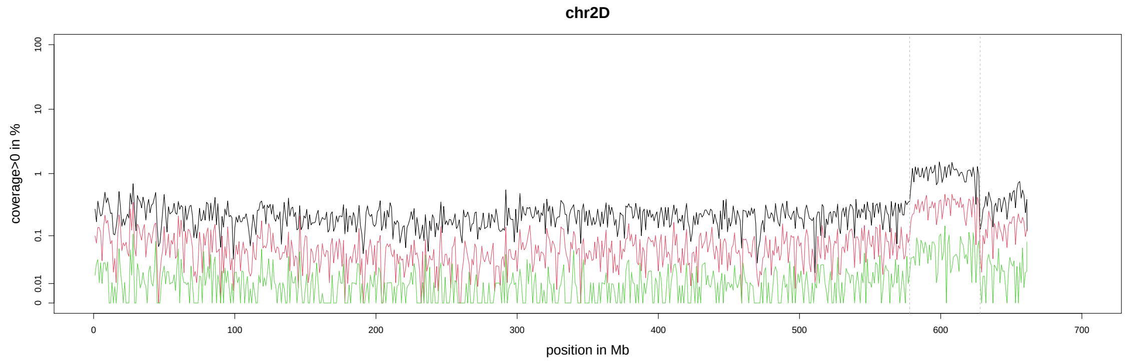

Fig. S2. Coverage profile of *Aegilops markgrafii* based on different depths of genotyping-by-sequencing (GBS) data. Trimmed reads of *Ae. markgrafii* (PI 596287) were mapped against cv. Julius and subsampled: 100% (black), 10% (red), and 1% (green). The introgression on chromosome 2DL was still detected when using only 10% and 1% of the data.

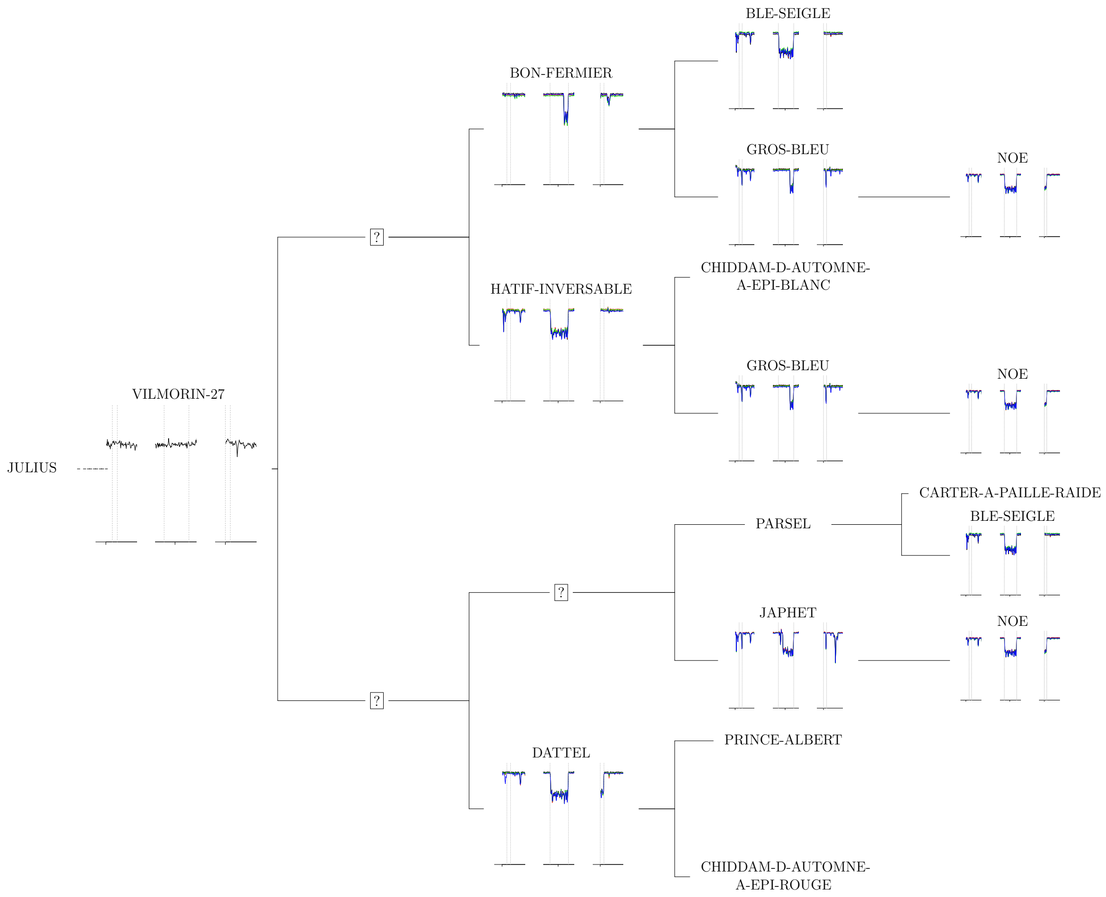

Fig. S3. Pedigree of cv. Vilmorin-27 augmented with the coverage profiles of the potentially introgressed regions on chromosomes 2AS (left, introgression 2), 2DL (middle, introgression 6), and 3DS (right, introgression 7). Available genotyping-by-sequencing (GBS) and whole genome sequencing (WGS) data for these cultivars were mapped against cv. Julius. Dashed lines mark the borders of the potentially introgressed regions. Different colours indicate different accessions used in analysis.

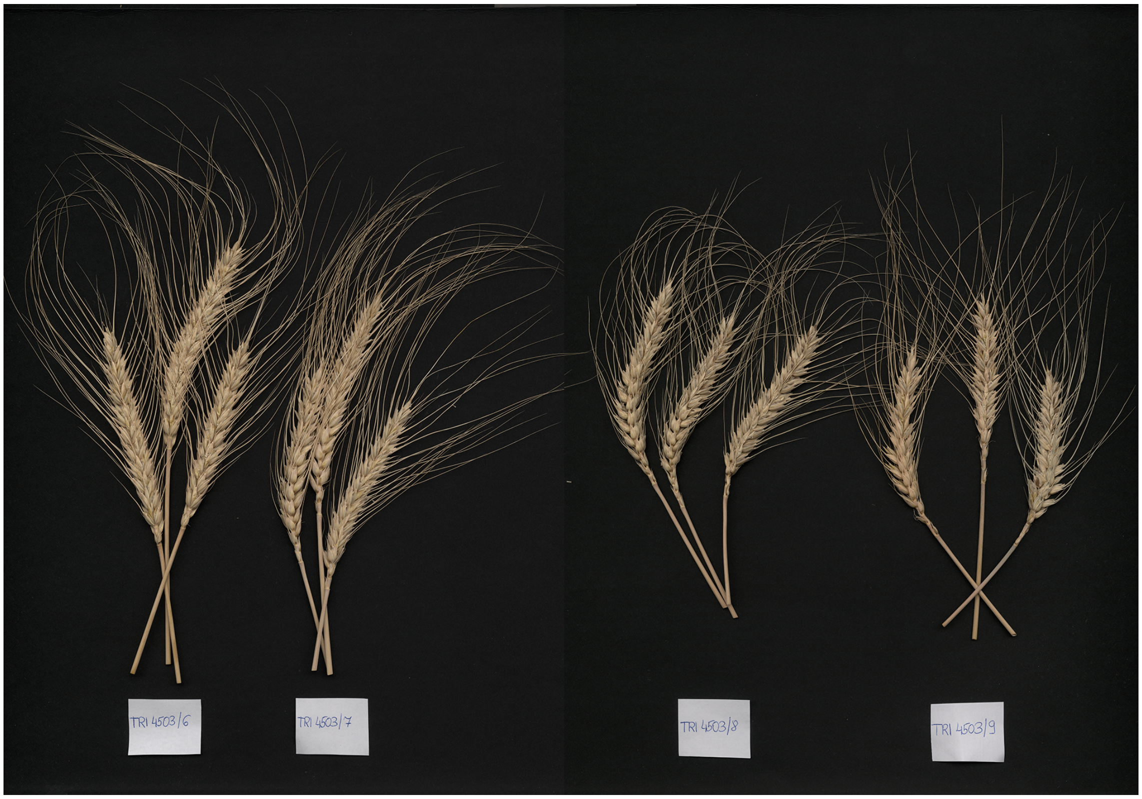

Fig. S4. Comparison of spikes from four mature plants of the old cultivar Krymka. No obvious differences in spike characteristics or other phenotypic traits were detected among individuals.

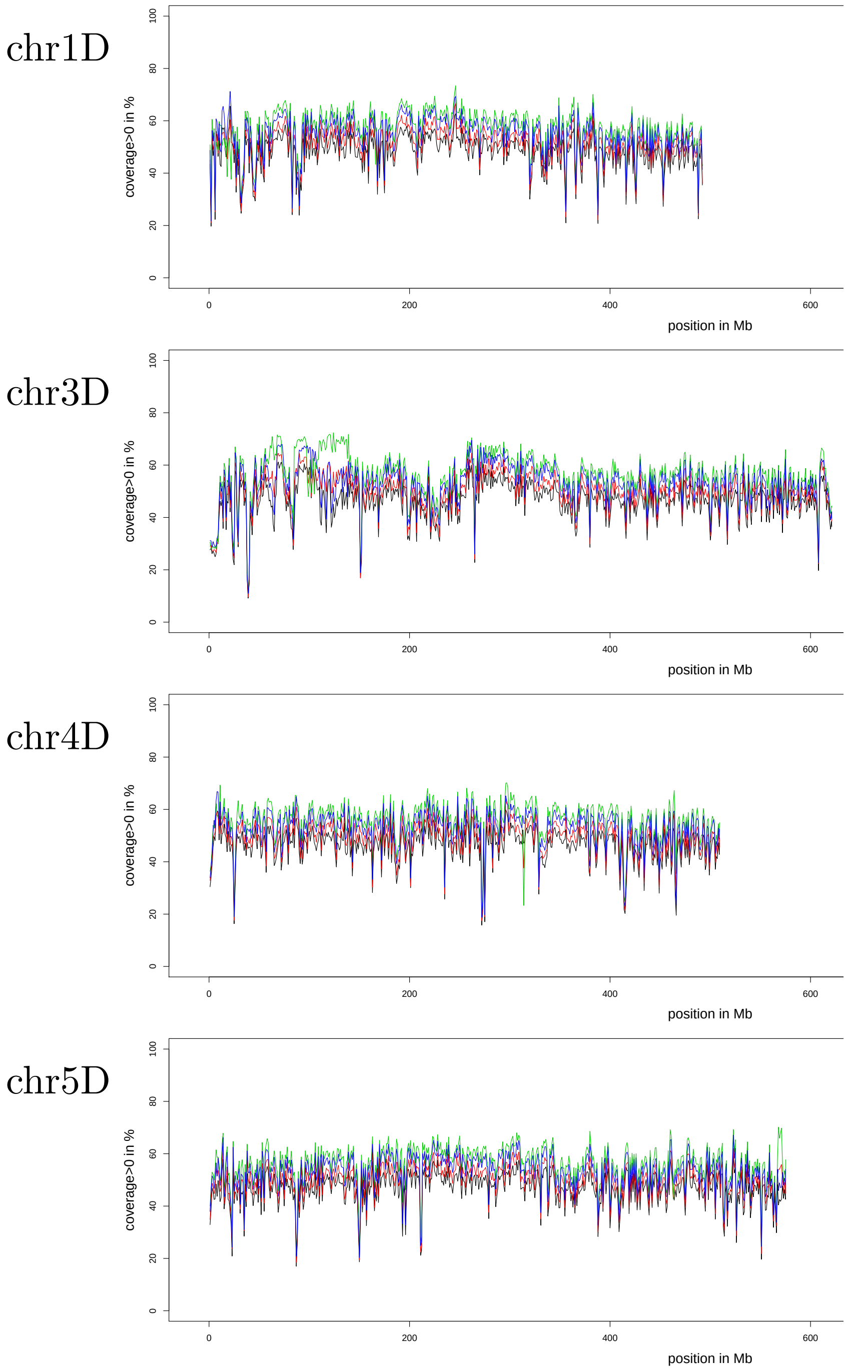

Fig. S5. Comparison of coverage profiles for four plants of AE 656. Coverage profiles of 4 individual plants of genebank accession AE 656 are depicted in four colors for chromosomes 1D, 3D, 4D and 5D using cv. Julius as reference genome. The plant visualized by the green profile clearly distinguishes from the other plants. The most prominent difference can be found on chromosome 3DS and 5DL.
